# Chemotherapy‐Induced Skeletal Muscle Dysfunction and Cognitive Impairment: A Muscle‐Brain Mechanistic Framework With Implications for Exercise Oncology

**DOI:** 10.1002/jcsm.70394

**Published:** 2026-10-07

**Authors:** SoYoung Ahn, Jin‐Soo Kim, Daniel A. Galvao, Dennis R. Taaffe, Dong Hyun Yoon, Carolyn McIntyre, Christelle Schofield, Yoon Jung Chang, Jihee Min, Wook Song, Robert U. Newton

**Affiliations:** ^1^ Institute of Sports Science, Department of Physical Education Seoul National University Seoul Republic of Korea; ^2^ Exercise Medicine Research Institute Edith Cowan University Joondalup Western Australia Australia; ^3^ School of Medical and Health Sciences Edith Cowan University Joondalup Western Australia Australia; ^4^ Department of Rehabilitation Medicine, College of Medicine Seoul National University Seoul Republic of Korea; ^5^ Institute on Aging Seoul National University Seoul Republic of Korea; ^6^ National Cancer Survivorship Center, National Cancer Control Institute National Cancer Center Goyang Republic of Korea; ^7^ School of Human Movement and Nutrition Sciences University of Queensland St Lucia Queensland Australia

**Keywords:** cancer, chemotherapy, cognitive impairment, inflammation, myokine, oxidative stress, skeletal muscle dysfunction

## Abstract

Chemotherapy commonly induces skeletal muscle dysfunction and cognitive impairment, complications that reduce treatment tolerance and quality of life. Skeletal muscle mass frequently declines during chemotherapy, with cohort studies reporting losses of 3%–10% across treatment courses, while chemotherapy‐induced cognitive impairment affects up to one‐third of patients. Emerging evidence indicates that chemotherapeutic agents impair mitochondrial function in peripheral tissues, including skeletal muscle, increasing systemic oxidative stress and inflammatory cytokines. These factors may promote oxidative stress and inflammation within the brain, particularly in the hippocampus, contributing to neuronal mitochondrial dysfunction, apoptosis and cognitive decline. Skeletal muscle may therefore represent an important component within a broader muscle–brain axis linking chemotherapy‐induced metabolic dysfunction with brain health. Targeted exercise improves skeletal muscle mitochondrial function, reduces systemic inflammation and oxidative stress and modulates multiple exercise‐responsive signalling pathways, including myokines such as brain‐derived neurotrophic factor and irisin. Collectively, these adaptations may contribute to improved neuronal health, neurogenesis and cognitive function. Understanding these interconnected mechanisms may inform exercise strategies to mitigate chemotherapy‐related cognitive impairment.

## 1 Introduction

Because of the growing burden of cancer incidence and mortality [1], the development of safe and effective cancer therapies has been prioritized, with various types of therapy applied alone or in combination, demonstrating substantial clinical improvement in controlling cancer [2]. In particular, the use of chemotherapy has been projected to increase over the next 20 years with its high efficacy on cancer control, and between 2018 and 2040, the number of patients with cancer requiring first‐course chemotherapy annually is estimated to increase from 9.8 million to 15 million [3]. However, these chemotherapeutic drugs result in various long‐term adverse effects impacting overall quality of life and survival of patients [4, 5].

Among these adverse effects, a reduction of skeletal muscle health and physical function among patients with cancer undertaking chemotherapy is now an emerging concern due to the negative impact on survival [6, 7]. Loss of skeletal muscle during systemic chemotherapy is increasingly recognized as a clinically significant complication and has been associated with poorer survival outcomes across several cancer types. Specifically, loss of skeletal muscle during neoadjuvant chemotherapy in patients with cancer is associated with survival and recurrence after adjuvant therapy [8, 9, 10]. In addition, negative effects of chemotherapeutic drugs on cognitive function have also been reported [11, 12, 13, 14]. A recent meta‐analysis revealed that one in three patients with breast cancer undertaking chemotherapy develop clinically significant cognitive impairment [13], while animal studies also showed alterations in objective neurological measures with subsequent reductions of cognitive function, indicating negative impact of chemotherapy on cognitive function [15, 16].

Recently, a hypothesis for chemotherapy‐induced cognitive impairment (CICI) involving peripheral oxidative stress and inflammation, causing severe endogenous oxidative stress and inflammation in the brain has emerged [17, 18]. It has been shown that chemotherapeutic drugs can induce mitochondrial dysfunction in peripheral tissues, which may lead to a severe increase in oxidative stress and pro‐inflammatory cytokines in the circulatory system [19, 20, 21]. This may create a feedback loop that amplifies endogenous inflammation in the hippocampus, causing substantial reactive oxygen species (ROS) production, further increasing endogenous inflammation [19, 20, 21]. The peripheral‐endogenous inflammation/ROS production feedback loop in the brain inevitably affects neuronal mitochondrial function, leading to apoptosis and degeneration of neuronal cells [17, 18].

It has been shown that exercise could mitigate systemic oxidative stress and inflammation through an anti‐oxidative function [22, 23, 24]. Moreover, skeletal muscle is recognized as an endocrine organ that produces cytokines (myokines), with exercise acting as a potent modulator of myokine secretion [22, 24]. As 40% of the body mass of healthy‐weight people consists of skeletal muscle [25], skeletal muscle is known to have a critical role in maintaining health through its endocrine function [22, 23, 24, 26]. Myokines also regulate adipogenesis and reduce chronic low‐grade systemic inflammation, preventing the development of chronic disease [22, 23, 24]. Specifically, the myokines BDNF and irisin are known to have a substantial impact on mitochondria, systemic inflammation and neuronal regeneration [27, 28], suggesting the potential involvement of myokines in ameliorating CICI. In this review, we provide an overview of clinical evidence and uncover the mechanistic link between skeletal muscle health dysfunction and cognitive impairment in patients undertaking chemotherapy. Moreover, we also discuss the potential endocrine role of skeletal muscle in muscle–brain interaction to provide clinical implications when tailoring exercise medicine for patients experiencing or at risk of CICI.

## 2 Impact of Chemotherapy on Skeletal Muscle Health

Skeletal muscle loss has become an emerging concern in patients with cancer [29, 30]. Specifically, for patients undertaking chemotherapy, an association between loss of skeletal muscle mass during chemotherapy and reduced survival and increased recurrence of cancer has been reported [31, 32, 33]. For instance, in a study involving patients with metastatic colorectal cancer undertaking chemotherapy, lower survival in patients who lost more than 9% of skeletal muscle mass during chemotherapy was shown, with skeletal muscle loss independently associated with survival [31]. In addition, patients with breast cancer receiving neoadjuvant chemotherapy who lost more than 3% of their skeletal muscle index showed poorer disease‐free survival than those who maintained skeletal muscle mass [32, 33], stressing the importance of skeletal muscle in patients with cancer undertaking chemotherapy [33].

Chemotherapeutic agents comprise a heterogeneous group of therapies that can be broadly classified according to their mechanisms of action, including alkylating agents (e.g., cyclophosphamide), antimetabolites (e.g., 5‐fluorouracil and methotrexate), microtubule‐targeting agents (e.g., paclitaxel and docetaxel), platinum compounds (e.g., cisplatin and carboplatin) and topoisomerase inhibitors (e.g., irinotecan) [34, 35]. Although these agents differ in their anti‐tumour mechanisms, cancer‐specific indications and treatment schedules, growing evidence suggests that many share common off‐target effects on healthy tissues, including skeletal muscle and the central nervous system. These adverse effects have been increasingly recognized as important contributors to treatment‐related declines in physical and cognitive function [34, 35, 36]. The tumour‐suppressive role of chemotherapeutic drugs involves various mechanisms. These drugs reduce DNA synthesis and increase DNA fragmentation, halting DNA replication and repair [2, 37, 38, 39, 40, 41, 42, 43, 44, 45, 46]. This results in reduced proliferation and invasion of cancer cells while increasing apoptosis [2]. However, the non‐specific cytotoxic action of chemotherapeutic drugs inevitably causes cellular damage to healthy somatic cells [47]. Moreover, as mitochondria contain their own DNA structures, mtDNAs, this non‐targeted mode of cytotoxic action affects cells with heavy metabolic demand [44]. Because of its high metabolic demand, mitochondrial density is greater in skeletal muscle compared with other somatic cells [48], and this makes skeletal muscle more vulnerable to the cytotoxic action of chemotherapeutic drugs [47].

In line with this, a number of retrospective studies and clinical trials showed a substantial loss of skeletal muscle mass and quality among patients during their course of chemotherapy (Table 1) [49, 50, 51, 52, 53, 54, 55, 56, 57, 58, 59, 60, 61, 62, 63, 64, 65, 66, 67, 68, 69, 70, 71, 77]. A significant reduction in skeletal muscle mass and skeletal lean mass index (lean mass adjusted for height^2^) has been reported in patients with lung cancer undertaking chemotherapy [62, 71], as well as patients with oesophageal cancer after neoadjuvant [65] and palliative chemotherapy [64]. Furthermore, neoadjuvant chemotherapy in patients with breast cancer significantly reduced muscle cross‐sectional area, skeletal muscle mass, muscle force and exercise capacity following chemotherapy [86, 87]. Importantly, biopsied samples of skeletal muscle from patients with breast cancer showed a significant reduction in mitochondrial function (measured by citrate synthesis and VDAC) with an increase of apoptotic cells after neoadjuvant chemotherapy, suggesting that chemotherapy may cause skeletal muscle atrophy and skeletal muscle mass loss through mitochondrial impairment [70]. This has been supported by a number of animal studies reporting the negative impact of chemotherapy on skeletal muscle mitochondria [51, 52, 53, 54, 55, 56, 57, 58, 59]. Specifically, animal studies in various cancer animal models showed impaired skeletal muscle mitochondrial function by demonstrating reduced NADH‐supported respiration and FADH_2_‐supported respiration while increasing H_2_O_2_‐emitting potential after chemotherapeutic drug injection [52, 56]. In addition, the reduction in mitochondrial biogenesis and mitophagy (e.g., PGC‐1α, PGC‐1β, Cytochrome C and Parkin) were observed in the skeletal muscle of chemotherapy‐treated animal models with a substantial increase of ROS production [52, 56, 57]. However, it was also evident that the well‐established muscle wasting‐associated markers, such as MuRF‐1, Fbxo21, Fbxo30, Fbxo 31, myostatin and Activin A, were unchanged in cancer animal models treated with chemotherapy [57], suggesting mitochondria may be a critical target for improving skeletal muscle health in patients undertaking chemotherapy. Recent reviews have highlighted chemotherapy‐induced myotoxicity as an underrecognized consequence of systemic cancer therapy, emphasizing mitochondrial dysfunction, oxidative stress, impaired protein turnover and metabolic dysregulation as central mechanisms contributing to skeletal muscle loss during treatment [34]. Emerging preclinical evidence suggests that post‐transcriptional regulation may also contribute to chemotherapy‐induced muscle wasting. In cachectic colon‐26 tumour‐bearing mice, 5‐fluorouracil exacerbated muscle loss via miR‐351‐3p‐mediated inhibition of ERK2 signalling, while suppression of this microRNA partially restored ERK phosphorylation and muscle fibre size [58, 88]. In addition to mitochondrial dysfunction, recent evidence suggests that anabolic resistance may represent another important mechanism contributing to chemotherapy‐associated muscle loss. Impaired anabolic signalling may reduce the capacity of skeletal muscle to respond to nutritional and exercise stimuli, thereby accelerating muscle wasting in patients with cancer [35].

**TABLE 1:** Chemotherapy‐induced skeletal muscle dysfunction and cognitive impairment: Preclinical and clinical evidence.

Author	Preclinical/clinical	Population/animal/cell	Experimental context	Treatment	Result
Skeletal muscle					
Angelino et al., 2025 [49]	Preclinical	BALB/c male mice (Control *n* = 7, C26 *n* = 7 for RNA‐seq analysis; additional cohorts used for mechanistic studies);	Tumour‐bearing (C26 cachexia model)	C26 adenocarcinoma inoculation	Body weight ↓; skeletal muscle mass ↓; Atrogin‐1 ↑; MuRF1 ↑; PGC‐1α ↓; PGC‐1β ↓; TFAM ↓; mitochondrial dysfunction ↑; oxidative phosphorylation ↓; cAMP‐PKA‐CREB1 signalling ↓
		C2C12 myotubes; human primary myotubes	Tumour‐conditioned medium exposure (in vitro)	C26/LLC tumour‐conditioned medium	Myotube atrophy ↑; cAMP production ↓; CREB phosphorylation ↓; PGC‐1α ↓; NOR1 ↓; PDE4B/PDE4D ↑
Gicquel et al., 2024 [50]	Preclinical	Male KIC PDAC mice (KIC *n* = 5, CTRL *n* = 7 for longitudinal MRI assessment; additional cohorts used for histology, transcriptomics, proteomics, and mitochondrial analyses)	Tumour‐bearing (spontaneous pancreatic ductal adenocarcinoma cachexia model)	Spontaneous PDAC development (KIC model)	Body weight ↓; gastrocnemius mass ↓; adipose tissue ↓; muscle fibre CSA ↓; MuRF1 ↑; muscle volume ↓; maximal force ↓; mitochondrial oxidative capacity ↓; PCr recovery time ↑; OXPHOS complex proteins ↓; mitochondrial DNA ↓; PGC‐1α ↓; TFAM ↓; mitochondrial structural abnormalities ↑; ROS‐related pathways ↑; glycolysis ↑; amino acid metabolism dysregulation ↑
Hain et al., 2019 [51]	Preclinical	Six‐week‐old Female athymic nude mice Group 1: Non‐Tumour; Group 2: MDA‐MB‐231 inoculation; Group 3: Non‐Tumour + Carboplatin; Group 4: MDA‐MB‐231 inoculation + Carboplatin	Tumour‐bearing + chemotherapy	Carboplatin 60 mg/kg/week, 4 days after tumour inoculation	Body weight ↓ Group 2, 3, 4 vs. Group 1; GAS weight: ↓ Group 2, 3, 4 vs. Group 1; TA weight: ↓ Group 2, 3, 4 vs. Group 1; EDL weight: ↓ Group 2, 3, 4 vs. Group 1; TA cross‐sectional area: ↓ Group 2, 3, 4 vs. Group 1; EDL specific force: ↓ Group 2, 3, 4 vs. Group
Gilliam et al., 2013 [52]	Preclinical	8–10 weeks old male Sprague–Dawley rats (*n* = 50)	Chemotherapy alone	Doxorubicin injection (20 mg/kg)	Body weight 48‐72 h ↓; Whole body fat 72 h ↓; Lean mass 72 h ↓; Whole‐body energy expenditure (oxygen consumption, carbon dioxide production, ambulatory activity, and calculated energy expenditure); gastrocnemius muscle: pyruvate‐supported respiration ↓, ADP‐stimulated respiration supported with pyruvate/malate ↓, mitochondrial NADH‐ and FADH2 supported respiration ↓, H_2_O_2_‐emitting potential ↑, calcium retention ↓
de Lima Junior et al., 2016 [53]	Preclinical	14 weeks old Wistar rats (*n* = 26)	Chemotherapy alone	Doxorubicin injection (15 mg/kg)	Body weight ↓; Muscle EDL/TA ratio ↓; EDL CSA ↓; serum: corticosterone ↑, testosterone ↓, insulin↑, glucose ↑, fat‐free acid ↑, adiponectin ↔; EDL: insulin sensitivity ↓, insulin tolerance ↓, glucose disappearance rate ↓, IRS‐1 ↓, GSK3‐b ↓, GULT 4 ↓, AMPK alpha ↓, mitochondrial complex 1 ↑, mitochondrial complex 3 ↓
Tarpey et al., 2019 [54]	Preclinical	10–24 weeks old C57BL/6NJ male mice (*n* = 41)	Chemotherapy alone	Doxorubicin injection (20 mg/kg)	Total body mass ↓; muscle mass corrected for body mass ↑; EDL & soleus muscle: muscle fibre CSA ↔, absolute force ↓, specific force ↓, percent initial force ↓, half relaxation time ↑, Ca^2^ + uptake rate ↓, mitochondrial oxygen consumption ↓, absolute respiratory sensitivity ↓, membrane potential ↓, pyruvate dehydrogenase kinase ↓
Gilliam et al., 2009 [55]	Preclinical	6–8 weeks old male TNFR1 receptor‐deficient mice	Chemotherapy alone	Doxorubicin injection (20 mg/kg)	EDL: muscle mass ↓, estimated CSA ↓, muscle force ↓, half relaxation time ↑, TNF mRNA ↑, TNF precursor protein ↑
Gouspillou et al., 2015 [56]	Preclinical	9 weeks old female C57BL/6 mice	Chemotherapy alone	Doxorubicin injection (10 mg/kg)	Body weight ↓; gastrocnemius muscle: mass ↓, fibre size (especially glycolytic muscle fibres, type IIx and type IIb) ↓, maximal respiratory capacity ↓, ROS emission ↑, free radical leak under state 3 driven by complex I and II substrates ↑
Barreto et al., 2016 [57]	Preclinical	8 weeks old CD2F1 male mice (*n* = 8)	Chemotherapy alone	5‐Fluorouracil‐leucovorin‐CPT‐11 cocktail, 5‐fluorouracil‐leucovorin‐oxaliplatin cocktail	Body weight ↓; body fat mass ↓; body lean mass ↓; quadriceps muscle: mass ↓; GAS: mass ↓; EDL: mass ↓, force ↓, mitochondria density ↓; TA: CSA ↓; whole‐body muscle force ↓; ERK1/2 phosphorylation ↑; p38 MAPK phosphorylation ↑; PGC‐1α ↓; PGC‐1β ↓; Cytochrome C ↓; succinate dehydrogenase activity ↓; oxidative fibre size ↓; glycolytic fibre size ↓; shift to glycolytic fibre from oxidative fibre in tibialis muscle; ROS ↑; Ucp1 ↓; Dideal ↓; Acot2 ↓; Fhl3↓; lipid metabolism and transport markers (Fabp1, Apoa 1, Apob, Apoa2, Alb, Prkcz, Scd2) ↑
		C2C12 murine myotubes	Chemotherapy exposure (in vitro)	5‐Fluorouracil‐leucovorin‐CPT‐11	Myotube atrophy ↑; MAPK ERK1/2 phosphorylation ↑; AKT ↓
Murphy et al., 2022 [58]	Preclinical	Male CD2F1 and Balb/c mice inoculated with C‐26 colon adenocarcinoma cell line	Tumour‐bearing + chemotherapy	5‐Fluorouracil (100 mg/kg)	Soleus muscle: mass ↓; extensor digitorum longus muscle: mass ↓; plantaris muscle: mass ↓; TA muscle: mass ↓; heart ↓; muscle fibre size (type IIx/b fibres) ↓; fibre type shift from type IIa fibres to type IIx/b fibre; miR‐351‐3p ↑; ERK1/2 Phosphorylation ↓
		C2C12 murine myotubes	Mechanistic intervention (in vitro)	miR‐351‐1p transfection	Myotube size ↓; ERK1/2 phosphorylation ↓
		C2C12 murine myotube	Chemotherapy exposure (in vitro)	Oxaliplatin, cisplatin, irinotecan	Oxaliplatin/Cisplatin treated: myotube size ↓; Irinotecan treated: ERK1/2 phosphorylation. ↑
Guigni et al., 2018 [59]	Clinical/preclinical	Patients with cancer: stage I, II, or III breast cancer undertaking chemotherapy (*n* = 13); healthy control (*n* = 12)	Patients with cancer receiving chemotherapy	Taxanes, Cyclophosphamide, Doxorubicin, Trastuzumab	Single‐muscle fibre CSA ↓; MHC I & II fibre CSA ↔; mitochondrial fractional area ↓; average mitochondrial area ↓; subsarcolemmal mitochondrial fractional area ↓; mitochondrial per unit area ↓; fractional content of MHC I proteins ↓; muscle oxidative stress (mitochondrial‐targeted antioxidant enzyme Prx3) ↑; tubulin and detryosinated tubulin ↑
		Cells: C2C12 murine myotube cell	Chemotherapy exposure (in vitro)	Dox/Taxol/Cis	Dox treated: Prx3 ↑; Dox and Taxol treated: mitochondrial content ↓, ROS production↑; Cis treated: myosin ↓
Kakinuma et al., 2017 [60]	Clinical	Stage IV NSCLC, scheduled to undertake chemotherapy (*n* = 65)	Patients with cancer receiving chemotherapy	Carboplatin, Pemetrexed, Bevacizumab, Cisplatin, Docetaxel cocktail	Skeletal muscle mass ↓
Guinan et al., 2018 [61]	Clinical	Patients with oesophageal cancer undertaking pre‐/post‐operative chemotherapy (*n* = 28)	Patients with cancer p receiving chemotherapy	Etoposide, Cisplatin, 5‐fluorouracil, Carboplatin, Paclitaxel	BMI ↔; SMI ↓; lean body mass ↓; lean tissue area at L3 ↓
Nattenmuller et al., 2017 [62]	Clinical	Patients with lung cancer receiving the first line of chemotherapy (*n* = 200)	Patients with cancer receiving chemotherapy	Carboplatin, Cisplatin, Gemcitabine, Vinorelbine, Etoposide, Pemetrexed	Total, volatile, saturated, intramuscular fatty acids ↑; ratio of volatile fatty acids to saturated fatty acids ↑; BMI ↓; muscle density ↓; muscle area ↓; skeletal muscle mass ↓
den Boer et al., 2020 [63]	Clinical	Patients with gastro‐oesophageal cancer undertook neoadjuvant chemotherapy before surgical resection (*n* = 199)	Patients with cancer receiving neoadjuvant chemotherapy	Epirubicin with/without Cisplatin, Capecitabine cocktail	SMI ↓; Skeletal muscle area ↓; number of patients with sarcopenia after the chemotherapy ↑
Dijksterhuis et al., 2019 [64]	Clinical	Patients with advanced oesophagogastric cancer treated with standard first‐line palliative chemotherapy (*n* = 88)	Patients with cancer receiving palliative chemotherapy	Capecitabine and Oxaliplatin (CapOx)	SMI ↓; sarcopenia obesity proportion ↑
Fujihata et al., 2021 [65]	Clinical	Patients with oesophageal cancer (stage <IV) treated with neoadjuvant chemotherapy	Patients with cancer receiving neoadjuvant chemotherapy	5‐Fluorouracil, Cisplatin, Docetaxel	SMI ↓; BMI ↓; psoas major muscle index ↓; side abdominal muscle index ↓; rectus abdominal muscle index ↓
Yoshino et al., 2020 [66]	Clinical	Patients with advanced epithelial ovarian cancer (stage III/IV) undertook chemotherapy (*n* = 75)	Patients with cancer receiving chemotherapy	Paclitaxel, Carboplatin, Docetaxel, Irinotecan	Skeletal muscle area ↓; number of patients with sarcopenia ↑; BMI ↓
Park et al., 2020 [67]	Clinical	Patients with advanced gastric cancer received chemotherapy (*n* = 111)	Patients with cancer receiving chemotherapy	5‐Fluorouracil, Leucovorin, Oxaliplatin, Capecitabine, Cisplatin cocktail	SMI ↓; body mass index ↓; weight ↓ (only in males)
Palmela et al., 2017 [68]	Clinical	Locally advanced adenocarcinoma from stomach or gastroesophageal junction received neoadjuvant chemotherapy (*n* = 48)	Patients with cancer receiving neoadjuvant chemotherapy	Mixture of epirubicin, cisplatin, 5‐fluorouracil, oxaliplatin, capecitabin, folinic acid docetaxel	Skeletal muscle area ↓; muscle radiation attenuation ↓; volatile fatty acids ↓; saturated fatty acids ↓; skeletal muscle index ↓; visceral fat index ↓
Cooper et al., 2015 [69]	Clinical	Patients with pancreatic ductal adenocarcinoma undertaking neoadjuvant chemotherapy (*n* = 82)	Patients with cancer receiving neoadjuvant chemotherapy	Gemcitabine and cisplatin	Skeletal muscle ↓; visceral adipose tissue ↓; subcutaneous adipose ↓
Mallard et al., 2024 [70]	Clinical	Patients with early breast cancer undergoing chemotherapy (*n* = 20)	Patients with breast cancer receiving chemotherapy	Epirubicin‐cyclophosphamide (EC) followed by paclitaxel (TAX)	Post EC: sedentary behaviour ↑, appetite level ↓, vastus lateralis muscle type I & IIa CSA↓; mitochondrial respiratory capacity (CI‐linked substrate state, OXPHOS by CI, CI & CII, CII) ↓, mitochondrial content marker (citrate synthase, VDAC) ↓, PGC‐1α1 ↑, mitochondrial inner‐membrane fusion marker (OPA1) ↓, PINK1 ↓, Mul1 ↓, antiapoptotic counterpart ↓, TUNEL‐positive signal ↑; post‐TAX: OXPHOS by CII ↑, catalase protein ↑, Bax ↔, TUNEL‐positive signal ↑
Stene et al., 2015 [71]	Clinical	Patients with non‐small cell lung cancer undergoing palliative chemotherapy (*n* = 35)	Patients with cancer receiving palliative chemotherapy	Carboplatin plus vinorelbine or carboplatin plus gemcitabine	Skeletal muscle CSA ↓; whole body muscle mass ↓
Cognitive function impairments					
Marques‐Aleixo et al. 2016 [72]	Preclinical	Male Sprague–Dawley rats	Chemotherapy alone	Doxorubicin (2 mg/kg)	Working memory ↓; activity and exploratory behaviour ↓; Hippocampus and cerebellum: mitochondrial respiration ↓, mitochondrial transmembrane electrical potential ↓, MDA level ↑, Mn‐SOD activity ↓, SIRT3↓, UCP2 ↓, Caspase 3 and 9 activities ↑, Bax/Bcl2 ratio ↑, cofilin ↑, PGC‐1α ↓, Mfn1↓, Mfn2 ↓, TFAM ↓, OPA1 ↓, DRP1 ↑, Beclin/Bcl2 ↑, LC3II ↑, Parkin ↓
Bagnall‐Moreau et al., 2019 [73]	Preclinical	Ovariectomised 8–10 weeks old female Sprague–Dawley rats	Chemotherapy alone	Cyclophosphamide (40 mg/kg) and Doxorubicin (4 mg/kg)	Hippocampus: inflammation markers ↑ (IFN‐gamma, IL‐2, IL‐3, IL‐4, IL‐6, IL‐10, LIX, L‐Selectin, MIG, MIP‐1α, MIP‐3α, VEGF, GF‐CSF, ICAM‐1, IL‐1ra, IP‐10, and TNF‐α), antioxidant and oxidative stress‐response gene ↑ (GPX1, NKkB, PRDX1, TNF‐α); Stress response signalling pathways ↑ (SAPK/JNK activation); (8‐OH(d)G DNA/RNA oxidation marker) ↑; ROS level ↑
Keeney et al., 2018 [15]	Preclinical	2–3 months old B6C3 (C57BL/6 x C3H hybrids, *n* = 6)	Chemotherapy alone	Doxorubicin (25 mg/kg)	Cognitive function: novel object recognition ↓, open field test ↓; brain tissue: protein carbonyls (damage to protein) ↑, protein‐bound HNE (damage to lipid) ↑; plasma: protein carbonyls ↑, protein‐bound HNE ↑; magnetic resonance spectroscopy (H^1^‐MRS): neuronal integrity (N‐acetylaspartate/creatine ratio) ↓; cell density and necrosis (choline‐containing compounds/creatine ratio) ↓; phosphatidylcholine‐specific phospholipase C and Phospholipase D activity ↓
Ramalingayya et al., 2016 [16]	Preclinical	26 weeks old female Wistar (*n* = 36)	Chemotherapy alone	Doxorubicin (2.5 mg/kg)	Object recognition test: recognition index ↓, Discriminative index ↓; hippocampal and cortex: TNF‐α ↑, oxidative stress (Catalase, GSH, thiols, SOD) ↑; serum: creatine kinase ↑, urea ↑, triglyceride ↑, total cholesterol ↑
		IMR32 cells	Chemotherapy exposure (in vitro)	Doxorubicin	Dox‐treated: cell viability ↓, cell cycle arrest at G_2_/M phase, neurite outgrowth ↓, apoptosis ↑; ROS ↑
Tong et al., 2020 [74]	Preclinical	6 weeks old Sprague‐Dowley rat (*n* = 24)	Chemotherapy alone	Doxorubicin (2 mg/kg)	Spatial memory ↓; short‐term memory ↓; hippocampus: nuclear pyknosis ↑, neuronal cell degeneration ↑, MDA (oxidative stress) ↑, GSH (oxidative stress) ↓, TNF‐α ↑, PGE‐2 ↑, COX‐2 ↑, NFkB ↑; Apoptosis ↑ (TUNEL, caspase‐3 activity, caspase‐9 activity)
Lv et al., 2020 [75]	Preclinical	Adult male Sprague Dawley rats (*n* = 48)	Chemotherapy alone	Methotrexate (100 mg/kg)	Spatial memory ↓; hippocampus: nuclear pyknosis ↑, caspase‐12 ↑, CHOP ↑, TUNEL‐positive cells ↑, DCX and Ki67 positive cells ↓
Renderio et al., 2016 [76]	Preclinical	12 weeks old ovariectomized female C57BL/6 J mice (*n* = 45)	Chemotherapy alone	Cyclophosphamide (80 mg/kg), doxorubicin (4 mg/kg), 5‐fluorouracil (40 mg/kg)	Body weight ↓; spatial learning ↓; spatial memory ↓; hippocampus: surviving cells ↓, neuronal differentiation ↓, Ki67 positive cells ↓
Huberts et al., 2024 [77]	Clinical	Patients with breast cancer (*n* = 503)	Patients with cancer receiving treatment	Endocrine therapy (*n* = 260); Chemo (immuno)therapy (*n* = 124); Radiotherapy (*n* = 267)	Cognitive function ↓; clinically relevant cognitive impairment: 21% → 38% → 32% (baseline, 6 months, 12 months); new‐onset cognitive impairment in 20% of patients; chemo (immuno)therapy associated with cognitive impairment risk (OR = 2.31)
Cerulla et al., 2019 [78]	Clinical	Patients with newly diagnostic breast cancer without metastasis (*n* = 51)	patients with breast cancer receiving chemotherapy	5‐Fluorouracil, epirubicin, cyclophosphamide and/or taxanes combination	Immediately after: Wechsler memory scale logical memory III ↓, semantic fluency ↓, FAS ↓, Trail making test‐A ↓, Trail making test—B ↓, Wechsler adult intelligence scale‐III letter (number sequencing) ↓, Wisconsin card sorting test ↓, Stroop test ↓; follow up (9 weeks): FAS ↓, Trail making test—A ↓, Trail making test—B ↓, Wechsler adult intelligence scale‐III letter (number sequencing) ↓, Wisconsin card sorting test ↓, Stroop test ↓
Cheng et al., 2013 [79]	Clinical	Patients with breast cancer underwent postoperative adjuvant chemotherapy (*n* = 40); healthy age‐matched control (*n* = 40)	Patients with Breast cancer receiving adjuvant chemotherapy	Doxorubicin, paclitaxel, cyclophosphamide, fluorouracil	MMSE ↓; verbal fluency test ↓; digital span ↓; event‐based prospective memory ↓; time‐based prospective memory ↔
Bedard et al., 2016 [80]	Clinical	Patients with Stage I and II breast cancer completed first course of adjuvant chemotherapy within 1 year (*n* = 80); Healthy age‐matched control (*n* = 80)	Patients with breast cancer receiving adjuvant chemotherapy	Fluorouracil, epirubicin, cyclophosphamide, docetaxel	Prospective memory: MIST summary score ↓; MIST accuracy ↓; 24 h delayed prospective memory ↓
Tong et al., 2020 [81]	Clinical	Patients with breast cancer diagnosed with invasive breast cancer (0‐II) without metastasis (*n* = 45) Group 1: patients with chemotherapy Group 2: patients without chemotherapy	Patients with breast cancer with/without chemotherapy	Common standard‐dose chemotherapy regimens	FACT‐Cog ↓; perceived cognitive impairments ↓; perceived cognitive abilities ↓; CDT ↓; MRI: N‐acetyl aspartate ↓, choline ↓, total creatine ↓
Merriman et al., 2017 [82]	Clinical	Age ≤ 75 postmenopausal patients with breast cancer (Stage I‐IIIA) receiving anastrozole Group 1: anastrozole (*n* = 158); Group 2: anastrozole + Chemotherapy (*n* = 104); Group 3: healthy postmenopausal control (*n* = 106)	Patients with breast cancer receiving endocrine therapy ± chemotherapy	Chemotherapy as needed	Memory ↓; language and communication ↓; sensorimotor function ↓ in Group 2 which persisted a year
Dhillon et al., 2018 [83]	Clinical	Patients with colorectal cancer (Group 1, localized *n* = 289; Group 2, metastatic or recurrent, *n* = 73) scheduled for first‐line of chemotherapy Healthy control (Group 3, *n* = 72)	Patients with colorectal cancer receiving chemotherapy	Chemotherapy as needed	Proportion of patients with ↓ total FACT‐cog and perceived cognitive impairment in patients with chemotherapy; Cog‐QOL ↓ in patients with chemotherapy
Skaali et al., 2011 [84]	Clinical	Patients with testicular cancer (*n* = 342) Group 1: patients receiving chemotherapy (*n* = 276); Group 2: patients receiving radiotherapy (*n* = 71)	Patients with testicular cancer receiving chemotherapy	Bleomycin, Etoposide, Cisplatin	3‐month post‐therapy Group 1 vs. Group 2: Emotional function ↓, role function ↓, social function ↓, number of patients with concentration problem ↑
Libert et al., 2016 [85]	Clinical	Group 1: Patients with hematologic malignancies scheduled to undertake chemotherapy (*n* = 89); Group 2: healthy subjects (*n* = 89)	Patients with hematologic cancer receiving chemotherapy	Chemotherapy as needed	Cognitive performance assessments ↓; perceived cognitive performances ↓

*Note:* The presented results are all significant (*p* < 0.05), with increases and reductions presented with arrows (↑ and ↓).

Abbreviations: ADP, adenosine diphosphate; BMI, body mass index; CDT, clock drawing test; CSA, cross‐sectional area; EDL, extensor digitorum longus; FACT‐Cog, functional assessment of cancer therapy‐cognitive function; FADH, flavin adenine dinucleotide; GAS, gastrocnemius; GLUT4, glucose transporter type 4; GSH, glutathione; IRS‐1, insulin receptor substrate 1; MDA, malondialdehyde; MIST, memory for intentions screening test; MMSE, mini‐mental state examination; Mn‐SOD, manganese superoxide dismutase; NADH, nicotinamide adenine dinucleotide; PGC‐1α, peroxisome proliferator‐activated receptor‐γ coactivator 1 α; QOL, quality of life; ROS, reactive oxygen species; SMI, skeletal muscle index; SOD, superoxide dismutase; TA, tibialis anterior; Ucp 1, uncoupling protein 1.

## 3 Impact of Chemotherapy on Cognitive Function

Negative impacts of chemotherapy on various domains of cognitive function have been consistently reported in clinical and preclinical studies (Table 1) [15, 16, 72, 73, 74, 75, 76, 78, 79, 80, 81, 82, 83, 84, 85]. For instance, patients with colorectal cancer showed a substantial reduction in total FACT‐Cog score after the commencement of chemotherapy compared with patients without chemotherapy [83]. Moreover, patients with hematologic malignancies treated with chemotherapy showed reduced Cognitive Performance Scores (CPS) calculated by assessing various tests examining domains of cognitive function (working memory, short‐term memory, processing speed/executive function/visual attention) compared with their healthy counterparts [85]. Similar impairment in various domains of cognitive function have been reported in patients with breast cancer as a decline in verbal attention/working memory, processing speed/executive function/visual attention, verbal memory, cognitive flexibility, interference, fluency and recognition after chemotherapy [78, 81]. Further, the MRS and DTI analysis supported the negative impact of chemotherapy on patients by showing reduced NAA and total creatine in both left and right posterior cingulate gyrus in patients with chemotherapy compared with non‐chemotherapy [81]. The association between measured metabolite levels and cognitive function was also observed, suggesting an independent chemotherapy‐specific cognitive impairment may be due to metabolite alteration in the brain [81].

Consistent with clinical data, various cancer animal models injected with chemotherapeutic drugs showed a reduction in cognitive function (Table 1) [15, 16, 72, 73, 74, 75, 76]. Following chemotherapy injection, a significant reduction in surviving neuronal cells and proliferation/differentiation markers while increasing nuclear pyknosis and apoptotic activity in the hippocampus with a substantial increase in inflammatory cytokines, such as TNF‐α, IL‐6, IL‐1ra and MIP‐1α has been shown [15, 16, 72, 73, 74, 75, 76]. Moreover, the increase in inflammatory cytokines in the hippocampus were accompanied by increased oxidative stress‐response genes (e.g., GPX1, NFκB, PRDX1), oxidative stress markers (e.g., MDA, Mn‐SOD, GSH and SOD) and DNA/RNA oxidative marker 8‐OH(d)G [73, 74]. This resulted in increased TUNEL and caspase‐3/9 activity, supporting the impact of chemotherapy‐induced oxidative stress and inflammation on neuronal survival [15, 16, 74]. Furthermore, the observed negative impact of chemotherapy on neuronal mitochondria in pre‐clinical work suggests the potential involvement of mitochondria impairment in cognitive impairment [72]. In this study, it was shown in the animal model injected with doxorubicin that the hippocampus and cerebellum mitochondria respiration and mitochondrial transmembrane electrical potential were substantially impaired [72]. This resulted in increased oxidative stress (MDA levels) and decreased anti‐oxidative activity (Mn‐SOD activity) in neuronal mitochondria [72], which lead to enhanced ROS production and protein carbonylation [89]. Mitochondrial biogenesis and mitophagy regulators, such as PGC‐1α, Mfn1, Mfn2, Parkin and Beclin 1, were also reduced with chemotherapy injection, leading to an increase in mitochondrial proteins involved in apoptotic signalling [72].

While a negative impact of endogenous inflammation and oxidative stress in the hippocampal area has been shown, many commonly used chemotherapeutic agents have limited ability to cross the blood–brain barrier, impeding chemotherapeutic drugs from directly influencing neuronal cells [90]. As such, peripheral oxidative stress and inflammation, contributing to increasing endogenous oxidative stress and inflammation resulting in CICI, have been proposed [17, 18, 20, 72, 91]. In animal models with chemotherapeutic drug injection, increased TNF‐α and creatine kinase in both plasma and the hippocampus area have been shown with increased oxidative stress markers protein carbonyl and protein‐bound HNE [15, 16]. With this increase in systemic and endogenous inflammation and oxidative stress markers, H^1^‐MRS showed a significant reduction in neuronal integrity (NAA/tCr ratio) and an increase in necrosis (Cho/tCr ratio), confirming the clinical observations [15, 81]. This suggests that a severe increase in pro‐inflammatory cytokines in the circulatory system caused by chemotherapy would negatively impact mitochondria in the brain [19, 20, 21]. This feedback loop connecting peripheral inflammation and mitochondrial dysfunction‐induced oxidative stress further creates a pro‐inflammatory and oxidative environment that causes apoptosis and neural degeneration, leading to a deleterious impact on cognitive function [19, 20, 21, 72].

## 4 Exercise in Patients Receiving Chemotherapy

Numerous exercise trials have been conducted in patients with cancer, and exercise has been highlighted as an important component of cancer management for both physical and psychological health benefits [33]. Moreover, improved skeletal muscle health and oxidative stress through enhanced mitochondrial function in various chemotherapy models were reported in clinical and preclinical studies, suggesting a potential link between skeletal muscle health and cognitive function during chemotherapy. Notably, exercise trials in patients with cancer, especially those undertaking chemotherapy, have resulted in substantial improvements in skeletal muscle health and physical function (Table 2) [92, 93, 94, 95, 96, 97, 98, 99, 100, 101, 102, 103, 104, 105, 106]. Specifically in patients with ovarian [99], gastrointestinal [100] and breast cancer [93, 98] undertaking various regimens of chemotherapy, a consistent increase in skeletal muscle mass, appendicular muscle mass index, muscular strength, physical function and maximal oxygen uptake (VO_2_ max) has resulted from exercise interventions [93, 96, 97, 98, 99, 100, 101, 106]. Given that chemotherapy‐induced skeletal muscle health impairment may be due to the untargeted action of chemotherapy drugs on mitochondria [19, 20, 21], improved VO_2_ max in patients with chemotherapy should be noted, as there is a linear relationship between VO_2_ max and skeletal muscle mitochondria content [116]. The improvement of VO_2_ max may be an indicator of improved muscle mitochondrial function and content, thereby positively impacting systemic inflammation and oxidative stress [116].

**TABLE 2:** Effect of exercise on muscle health and cognitive function in chemotherapy‐treated subjects.

Author	Preclinical/clinical	Population/animal/cell	Treatment	Exercise	Result
Skeletal muscle health					
Park S et al., 2019 [92]	Preclinical	Male 8 weeks old Sprague–Dawley rats Group 1: control; Group 2: control + EX; Group 3: doxorubicin; Group 4: doxorubicin+EX	2 mg/kg of doxorubicin	4‐week treadmill running without electrical stimulation (30 min at 10 m/min for 6 days/week)	Muscle strength ↑; fatigue index ↓; Dorsal raphe nucleus: number of tryptophan hydroxylase positive cells ↓, number of 5‐hydroxytryptamine positive cells ↓; GAS: Bax ↓, Bd‐2 ↑, caspase‐3 ↓, mitochondrial O_2_ respiration ↑ (adenosine diphosphate infusion, succinate infusion), mitochondrial H_2_O_2_ emission ↑ (glutamate & malate infusion, glutamate, malate, &, succinate infusion, glutamate, malate, succinate, and glycerol 3‐phosphate infusion), mitochondrial Ca^2^ retention capacity ↑
Courneya et al., 2007 [93]	Clinical	Patients with breast cancer initiating adjuvant chemotherapy (*n* = 242) Aerobic exercise (AET, *n* = 78); resistance exercise (RET, *n* = 82); usual care (*n* = 82)	Chemotherapy (fluorouracil, epirubicin, cyclophosphamide; fluorouracil; doxorubicin, paclitaxel)	Aerobic (3 days/week, 60%–80% VO_2_ max 15–45 min) Resistance (3 days/week, 2 sets of 8–12 repetitions, 60%–70% 1RM)	AET: Self‐esteem ↑, VO2 peak ↑, 1RM leg ↑, 1RM chest ↑; RET: Self‐esteem ↑, 1RM leg ↑, 1RM chest ↑, Lean mass ↑
Courneya et al., 2013 [94]	Clinical	Patients with breast cancer (*n* = 301) Standard aerobic exercise group (STAN, *n* = 96); high‐intensity exercise group (HIGH, *n* = 101); combined exercise group (COMB, *n* = 104)	Adjuvant chemotherapy (doxorubicin, cyclophosphamide, paclitaxel, adriamycin, herceptin, fluorouracil, epirubicin)	16‐week 3 days/week STAN: 25–30 min aerobic (55%–75% VO_2_ peak) HIGH: 50–60 min aerobic (55%–75% VO_2_ peak) COMB: 50–60 min aerobic (25–30 min 55%–75% VO_2_peak) + resistance (30–35 min 60%–75% 1RM)	Physical component summary: HIGH ↑ (vs STAN); Bodily pain: High ↑ (vs STAN and COMB); Endocrine symptoms: HIGH, COMB ↑ (vs STAN); Upper body strength: COMB ↑ (vs STAN, HIGH); Lower body strength: COMB ↑ (vs STAN, HIGH); Upper body endurance: COMB ↑ (vs STAN, HIGH); Lower body endurance: COMB ↑ (vs STAN, HIGH); VO_2_peak: High ↑ (vs COMB)
Courneya et al., 2014 [95]	Clinical	Patients with breast cancer (*n* = 301) Standard aerobic exercise group (STAN, *n* = 95); high‐intensity exercise group (HIGH, *n* = 99); Combined exercise group (COMB, *n* = 102)	Chemotherapy Anthracyclines (*n* = 115), concurrent Taxane (*n* = 155), Sequential Taxane (*n* = 201)		[Healthy weight subgroup] bodily pain: STAN ↓ (vs HIGH); endocrine symptoms: STAN ↓ (vs HIGH, COMB); taxane symptoms: STAN ↓ (vs HIGH), COMB ↓ (vs High), muscular strength: COMB ↑ (vs STAN, HIGH), STAN ↑ (vs High) [overweight/obese subgroup] muscular strength: COMB ↑ (vs STAN, HIGH) [obese subgroup] fat mass: COMB ↓ (vs HIGH) [premenopausal subgroup] bodily pain: STAN and COMB ↓ (vs HIGH); endocrine symptoms: STAN ↓ (vs HIGH, COMB); taxane symptoms: STAN ↓ (vs HIGH) [age < 50 subgroup] bodily pain: STAN and COMB ↓ (vs HIGH); taxane symptoms: STAN ↓ (vs HIGH) [Fit subgroup] endocrine symptoms: STAN ↓ (vs HIGH, COMB); taxane symptoms: STAN ↓ (vs HIGH, COMB)
Mijwel et al., 2019 [96]	Clinical	Breast cancer stage I‐IIIa (*n* = 206) Resistance HIIT (RT‐HIIT, *n* = 74); aerobic HIIT (AT‐HIIT, *n* = 72); usual care (UC, *n* = 60)	Anthracyclines, taxanes, or a combination	16‐week high‐intensity interval training (3 × 3min/day for 2 days/week; high intensity at RPE: 16–18)	RT‐HIIT: cancer‐related fatigue ↑, total and physical symptom ↑, handgrip strength ↑, lower‐limb muscle strength ↑; AT‐HIIT: cancer‐related fatigue ↑, total and physical symptoms ↑, hand grip strength ↑, lower‐limb muscle strength ↑, body mass↓; UC: VO_2_ peak ↓
Mijwel et al., 2018 [97]	Clinical	Breast cancer stage I‐IIIa (*n* = 23) Resistance HIIT (RT‐HIIT, *n* = 7); aerobic HIIT (AT‐HIIT, *n* = 6); usual care (UC, *n* = 10)	Anthracyclines, taxanes, or a combination		RT‐HIIT: mitochondrial content ↑ (citrate synthase activity), CSA type I & type IIA ↑, SC count per fibre ↑, capillaries per muscle fibre ↑, myosin heavy chain type I ↑; AT‐HIIT: mitochondrial content ↑ (citrate synthase activity, oxphos complexes), CSA type I ↑, capillaries per muscle fibre ↑, PINK1 (mitophagy marker) ↑; UC: mitochondrial content (citrate synthase activity) ↓, CSA type I & type IIA ↓, capillaries per muscle fibre ↓, myosin heavy chain type I ↓, PINK1 ↓, SOD ↑
Cešeiko et al., 2020 [98]	Clinical	Newly diagnosed Stage I–III breast cancer (*n* = 55) Maximal strength training group (*n* = 27); control group (*n* = 28)	Adjuvant therapy (doxorubicin, epirubicin, cyclophosphamide; some of paclitaxel or docetaxel; HER2‐positive tumours trastuzumab after chemotherapy)	12‐week horizontal leg press (20 min/day for 2 days/week; 4 sets 4 reps at 90% of 1RM)	Exercise group: Leg press 1RM ↑, time to exhaustion in treadmill test ↑, 6‐min walking test ↑, chair rising performance ↑, stair climbing performance ↑; control group: thigh volume ↓, quadriceps femoris muscle mass ↓, 6‐min walking test ↓, chair rising performance ↓, stair climbing performance ↓
Schofield et al., 2023 [99]	Clinical	Ovarian cancer survivors (*n* = 15)	Neoadjuvant (*n* = 10) Adjuvant (*n* = 5)	12‐Week supervised (in‐clinic or by telehealth) resistance exercise (50–60 min/day for 2 days/week; moderate intensity at RPE 13)	Body weight ↑; whole body lean mass ↑; appendicular lean mass ↑; muscle density ↑; upper and lower body strength ↑; 400 m walk test ↑; timed up and go ↑
Stuecher et al., 2018 [100]	Clinical	Gastrointestinal cancer and a locally or systemically advanced disease stage UICC III‐IV (*n* = 44); intervention (*n* = 22); control (*n* = 22)	Neoadjuvant, adjuvant or palliative	12‐Week home‐based walking exercise (30 min/day for 5 days/week; moderate intensity at RPE: 11–13)	Intervention: postural sway (centre of pressure) ↓, lean body mass ↑; Control: SPPB ↓
Allen et al., 2021 [101]	Clinical	Locally advanced oesophagogastric cancer (*n* = 54); prehabilitation (*n* = 26); usual care (*n* = 28)	Neoadjuvant therapy	15‐Week supervised exercise program (cycling + resistance + flexibility; 60 min/day for 2 days/week; moderate intensity at RPE 12–14) + additional home exercises 3 days/week +6 sessions of psychological coaching	VO_2_ peak ↑; weekly step count ↑; handgrip strength ↑ (less decline 6 weeks post‐operation); global health status ↑
Yen et al., 2019 [102]	Clinical	Head and neck patients (*n* = 84) exercise group (*n* = 42); sedentary group (*n* = 42)	Chemotherapy (*n* = 81; Cisplatin, Carboplatin, Gemcitabine, Medroxyprogesterone, Uracil‐Tegafur); Target (*n* = 3; Cetuximab)	8‐Week aerobic & resistance exercise (aerobic: 30 min/day 3 days/week at 60%–70% HRmax + resistance 3 sets ×10 reps at moderate intensity of RPE “somewhat heavy to heavy” using TheraBand)	6‐min walking test ↑; SBP ↓; DBP ↓; rHR ↓; resting mean arterial pressure ↓, heart rate recovery ↑; SpO_2_ ↑; rate‐pressure product ↑; RPE ↓; walking distance ↑
Yen et al., 2020 [103]	Clinical	Patients with head and neck cancer Exercise group (*n* = 30)	Chemotherapy (Cisplatin, Gemcitabine, Medroxyprogesterone)	8‐Week gym‐based aerobic & resistance (3 days/week age‐predicted HRmax 60%–70% 40–50 min, RPE somewhat heavy—heavy, 3 sets, 10–12 reps)	SBP ↓; DBP ↓; rHR ↓; Antioxidant capacity ↑; carbonyl ↓; 8‐Hydroxy‐2′‐deoxyguanosine ↓
Lin et al., 2014 [104]	Clinical	Patients with colorectal cancer Supervised exercise (*n* = 21); usual‐care (*n* = 24)	Adjuvant chemotherapy regimen (UFT, Oxali, 5‐FU, Capecitabine, 5‐FU/LV, Xeloda cocktail)	12‐Week aerobic & resistance (2 days/week, 30–40 min of cycling and whole‐body resistance exercise HRmax 40%–55% & RPE 11–12 ~ HRmax 60%–75% & RPE 14–15).	EORTC QLQ‐C30 ↑; 6‐m walk test distance ↑; handgrip strength ↑
Kleckner et al., 2019 [105]	Clinical	*n* = 293 Exercise group (*n* = 144) Control group (*n* = 149)	Cyclophosphamide + doxorubicin, Cyclophosphamide + Docetaxel/Paclitaxel, Carboplatin + Docetaxel/Paclitaxel, FOLFOX [Leucovorin, 5‐fluorouracil, Oxaliplatin], CMF [Cyclophosphamide, Methotrexate, 5‐fluorouracil], Carboplatin/Cisplatin/Oxaliplatin, R‐CHOP [Rituximab, Cyclophosphamide, Doxorubicin, Vincristine, and Prednisone]	6‐Week home‐based aerobic and resistance (aerobic: increase 5%–20% steps according to their baseline walking steps + resistance maximum 4 sets 15 reps low‐to‐moderate intensity of RPE “3–5”)	Exercise group: pro‐inflammation marker ↓ (IFNγ, IL‐8, IL‐1β), anti‐inflammation marker ↑ (IL‐6, IL‐10, sTNFR1); control group: IL‐8 ↓, IL‐10, sTNFR1 ↑
Adamsen et al., 2009 [106]	Clinical	Patients with cancer (*n* = 269) Control (*n* = 134) Intervention (*n* = 135)	Adjuvant chemotherapy	6‐Week multimodal intervention [Supervised 3 days/week, high intensity, 90 min (30 min warm‐up, 45 min resistance exercise, 15 min cardio) + Relaxation (30 min, 4 days/week), body awareness and restorative training (90 min, 1 day/week), and massage (30 min 2 days/week)]	Physical component scale ↑; VO_2_max ↑; leg press ↑; chest press; pull‐down ↑; EORTC QLQ‐C30 functional scale: fatigue ↓; General well‐being: role physical ↑, physical functioning ↑, vitality ↑, role emotional ↑, mental health ↑; mental component scale ↑
Cognitive function					
Park et al., 2018 [107]	Preclinical	Male 6 weeks old Wistar rats (*n* = 60) Group 1: control; group 2: EX; group 3: doxorubicin; group 4: doxorubicin + EX (*n* = 15 each)	2 mg/kg of doxorubicin	4‐Week treadmill running without electrical stimulation (30 min/day at 10 m/min for 6 days/week)	Group 3 (vs. group 1): spatial and working memory (Morris water maze) ↓, short‐term memory (step‐down avoidance task) ↓, hippocampal BDNF and TrkB ↓, hippocampal cell apoptosis (Bax ↑, Bcl‐2 ↑, cytochrome c ↑, cleaved caspase‐3 ↑); group 4 (vs group 3): short‐term memory ↑, spatial and working memory ↑, hippocampal BDNF and TrkB ↑, neurogenesis (BrdU/NeuN) ↑, Bax ↓, Bcl‐2 ↓, cytochrome c ↓, cleaved caspase‐3 ↓
Park et al., 2023 [108]	Preclinical	Male 8 weeks old Sprague–Dawley rats (*n* = 40) Group 1: control; Group 2: EX; Group 3: doxorubicin; Group 4: doxorubicin + EX (*n* = 10 each)	2 mg/kg of doxorubicin	8‐Week treadmill exercise (5 min at 3 m/min + 25 min at 8 m/min for 5 days/week)	Group 4 (vs. group 3): short‐term memory (step‐down avoidance test) ↑, TNF‐α ↓, IL‐6 ↓; Hippocampus: β‐catenin↓, Wnt ↓, BDNF ↑, TrkB ↑, Mitochondrial Ca^2^ retention capacity ↑
Lee et al., 2023 [109]	Preclinical	Male BALB/c mice Group 1: control; Group 2: EX; Group 3: sorafenib; group 4: sorafenib + EX	Sorafenib (60 mg/kg)	Voluntary wheel running (28 days)	Group 4 (vs. Group 3): body weight loss ↓, working memory ↑, anhedonia‐like behaviours ↓, Ki‐67 positive neurons (proliferating cell) ↑, DCX positive (immature neurons) ↑
Elbeltagy et al., 2024 [110]	Preclinical	6 Weeks old male mice Group 1: control; Group 2: cisplatin; Group 3: cisplatin + EX	Cisplatin (2.3 mg/kg/week)	14‐Week treadmill exercise (60 min, speed 14–19 m/min for 5 days per week)	Group 3 (vs. Group 2): preference index ↑, weight loss ↓, Ki‐67 positive neurons (proliferating cell) ↑, GPx positive (oxidative stress) ↓
Koevoets et al., 2023 [111]	Clinical	Breast cancer Stage I–III, 2–4 years after diagnosis (*n* = 181) Intervention group (*n* = 91); control group (*n* = 90)	Neoadjuvant chemotherapy	24‐Week aerobic & strength exercise (2 days per week, 60 min, 20 reps, 15 RM) + Nordic or power walking (2 days per week, 60 min, HRR 55%–65%)	VO_2_ peak ↑; Highly fatigued patients: VO_2_ peak ↑, total hippocampal volume ↓, right hippocampal volume ↓, right dentate gyrus ↓, and right subiculum ↓; positive association: hippocampal volume and HVLT‐R score, hippocampal volume and ACS wordlist learning performance
Campbell et al., 2017 [112]	Clinical	Postmenopausal I–IIIa breast cancer survivors (*n* = 19) Aerobic exercise (*n* = 10); delayed exercise (control, *n* = 9)	Chemotherapy (doxorubicin and cyclophosphamide; docetaxel and cyclophosphamide; 5‐fluorouracil, epirubicin, and cyclophosphamide)	24‐Week aerobic exercise (45 min/day supervised for 2 days/week +30 min/day home session for 2 days/week; moderate‐to‐vigorous intensity 60%–80% HRR)	VO_2_ peak ↑; objective neuropsychological (trail making test‐A) ↑; controlled oral word association test ↔; animals ↔; trail making test‐B ↔; trail making test‐Diff. ↔; Hopkins verbal learning test‐revised (memory function) ↔
Gokal et al., 2018 [113]	Clinical	Patients with breast cancer (*n* = 50) Self‐managed walking (*n* = 25); usual care (control, *n* = 25)	Neo‐adjuvant and adjuvant chemotherapy (fluorouracil, epirubicin and cyclophosphamide; followed by taxotere)	12‐Week home‐based, self‐managed intervention (10–30 min/day for 5 days/week; moderate intensity)	Working memory digit forward ↑, cognitive failures questionnaire ↑ (in usual care), Stroop task ↔, sustained attention to response task ↔, visuospatial skills ↔, working memory digit backward ↔
Salerno et al., 2019 [114]	Clinical	Breast cancer survivors (*n* = 27) Aerobic (*n* = 27); seated rest (*n* = 27)	Chemotherapy (82.1%)	30‐Min bout of moderate‐intensity aerobic exercise (40%–60% HRmax)	Cognitive battery: processing speed reaction time ↓; spatial working memory task: accuracy ↑, reaction time ↓
Northey et al., 2019 [115]	Clinical	Female cancer survivors (*n* = 17) High‐intensity interval training (HIIT, *n* = 6); moderate‐intensity continuous training (MOD, *n* = 5); wait‐list control (*n* = 6)	Chemotherapy, radiation therapy, hormonal therapy	12‐Week cycle exercise (3 days/week HIIT: 30s HR 90% < + 2 min recovery; MOD: 55% peak power)	HIIT: episodic memory ↑, working memory ↑, executive function ↑, cerebral blood flow ↑, cerebrovascular reactivity ↑, VO_2_ peak ↑

*Note:* The presented results are all significant (*p* < 0.05), and the increases and reductions are presented with arrows (↑ and ↓).

Abbreviations: ACS, Amsterdam Cognitive Scan; BDNF, brain‐derived neurotrophic factor; CSA, cross‐sectional area; DBP, diastolic blood pressure; DCX, doublecortin; EORTC QLQ, European Organisation for Research and Treatment of Cancer Quality of Life Questionnaire; GAS, gastrocnemius; GPx, glutathione peroxidase; HIIT, high‐intensity interval training; HRmax, maximum heart rate; HRR, heart rate reserve; HVLT‐R, Hopkins Verbal Learning Test‐Revised; OD, superoxide dismutase; rHR, resting heart rate; RM, repetition maximum; RPE, rate of perceived exertion; SpO_2_, saturation of peripheral oxygen; SPPB, Short Physical Performance Battery; SSBP, systolic blood pressure; TNF‐α, tumour necrosis factor‐alpha; TrkB, Tropomyosin receptor kinase B; VO_2_ peak, peak oxygen consumption.

Studies examining the role of exercise on skeletal muscle mitochondria in patients undergoing chemotherapy support the positive impact of exercise on muscle mitochondria [47]. For instance, in one randomized controlled trial, patients with breast cancer undertaking chemotherapy showed improved VO_2_ max after high‐intensity interval aerobic and resistance exercise with enhanced citrate synthesis activity and PINK1 in biopsied muscle samples, indicating enhanced mitochondrial biogenesis and quality [97]. Moreover, animal models support a positive impact of exercise on muscle mitochondria [92]. Accompanying an increase in skeletal muscle strength and fatigue index, the skeletal muscle of exercised animals with chemotherapy showed alteration of apoptotic signalling, such as increased Bax, and reduced Bcl‐2 and caspase‐3 activity [92]. Importantly, improvement of mitochondrial function has been evidenced by exercise with enhanced O_2_ respiration, improved mitochondrial Ca^2+^ retention and reduced ROS production (reduced H_2_O_2_ emission) [92]. Moreover, recent randomized controlled trials in patients with cancer undertaking chemotherapy further support the positive impact of exercise on systemic inflammation and oxidative stress by showing a significant reduction of plasma levels of protein carbonyl, 8‐OHdG, IFN‐γ and IL‐1β with prolonged‐combined exercise (aerobic and resistance) programs [103, 105], suggesting a positive impact of exercise on skeletal muscle mitochondria, which may mitigate systemic inflammation and oxidative stress.

As with the positive impact of exercise on skeletal muscle health, including an increase in skeletal muscle mass and enhanced mitochondrial function and quality, the positive role of exercise on cognitive function has also been demonstrated in multiple preclinical and clinical studies (Table 2) [107, 108, 109, 110, 111, 112, 113, 114, 115, 117]. In randomized controlled trials in cancer patients undertaking chemotherapy, consistent improvements in various domains of cognitive function, including working memory, processing speed, executive function and visual attention, as well as self‐reported cognitive function after prolonged combined exercise intervention, have been reported [111, 112, 113, 114, 115, 117]. Importantly, it appears that exercise may have a more profound impact on patients undertaking chemotherapy who experience high fatigue compared with those who do not [111, 117]. However, a rather unexpected negative association between hippocampal volume and cognitive function was also reported after long‐term exercise in patients with cancer undertaking chemotherapy and experiencing severe fatigue [111, 117]. It is known that inflammation is increased in patients undertaking chemotherapy and is associated with fatigue [118]; cellular swelling due to inflammation, which may contribute to the increased hippocampus volume, might therefore have been attenuated by prolonged exercise through its anti‐inflammatory effect [111, 118].

The positive effect of exercise in CICI is further supported in a number of animal models injected with chemotherapeutic drugs [72]. Importantly, animal studies found that an improvement of neuronal mitochondrial function and quality, which can reduce inflammation and oxidative stress, may have a positive impact on cognitive function [107, 108, 109, 110]. With improved working memory, short‐term memory and preference index, the exercised animals also showed a substantial improvement in hippocampus and cerebellum mitochondria regulatory genes for function and mitophagy, such as SIRT, UCP2, PGC1α, Mfn1, Mfn2, TFAM, Parkin, DRP1 and TrkB [107, 108, 109, 110]. This resulted in improved mitochondrial O_2_ consumption, mitochondrial PTP sensitivity and improved antioxidant activity (Mn‐SOD activity) while reducing ROS production (H_2_O_2_ emission), oxidative damage (MDA levels) and inflammatory markers (e.g., TNF‐α and IL‐6) in both plasma and the brain [107, 108, 109, 110], suggesting a potential link between the anti‐oxidative and anti‐inflammatory function of exercise and improvement of cognitive function [108].

## 5 Potential Role of Myokines Within the Muscle–Brain Axis During Chemotherapy

Mitochondrial dysfunction, oxidative stress, systemic inflammation and metabolic abnormalities are increasingly recognized as central mechanisms underlying both chemotherapy‐induced skeletal muscle dysfunction and cognitive impairment [34, 35]. Within this broader pathophysiological framework, alterations in skeletal muscle endocrine function, including myokine secretion, may represent one potential pathway through which peripheral muscle health influences brain function [119].

Importantly, these mechanisms are highly interconnected. Chemotherapy‐induced mitochondrial dysfunction can increase ROS production and impair cellular energy metabolism, thereby promoting systemic inflammation and metabolic dysregulation [56, 57, 120]. These alterations contribute not only to skeletal muscle wasting and weakness but also to neuroinflammation, neuronal dysfunction and cognitive decline. Therefore, rather than acting as primary upstream regulators, myokines should be considered as one component within a complex biological network linking skeletal muscle health and brain function during chemotherapy [119].

In the early 2000s, skeletal muscle was identified as an endocrine organ that synthesizes and secretes myokines into the circulatory system, and these myokines have a predominant impact on maintaining metabolic homeostasis and preventing chronic disease [22, 23, 24]. Exercise exerts pleiotropic effects on multiple biological systems involved in chemotherapy‐induced toxicities. In addition to altering myokine secretion, exercise improves mitochondrial function, reduces oxidative stress, enhances insulin sensitivity and attenuates chronic inflammation [22, 23, 24, 33, 34, 35]. Consequently, the beneficial effects of exercise on cognitive function are unlikely to be attributable to a single pathway.

Specifically, myokines have a substantial role in exercise‐derived adaptation, including enhanced mitochondrial health (enhanced mitochondria contents, increased mtDNA contents and facilitation of mitophagy), and have a profound impact on reducing peripheral oxidative stress and systemic inflammation [S1, S2]. As such, given that skeletal muscle comprises 40% of whole body mass [25] with high‐mitochondrial density [48], exercise‐induced myokines may contribute to improvements in muscular mitochondrial function and cognitive function through interactions with anti‐oxidative and anti‐inflammatory pathways (Figure 1) [22, 23, 24]. Importantly, preclinical studies have indicated the positive role of myokines BDNF, FGF‐21, IL‐6, IL‐15, irisin and SPARC in mitochondrial biogenesis and mitophagy [S3]. Several exercise‐responsive myokines, including FGF‐21, IL‐6, IL‐15, SPARC, oncostatin M and decorin, have been implicated in mitochondrial biogenesis, metabolic regulation, inflammation, tissue remodelling and tumour‐related pathways. Importantly, the physiological role of IL‐6 appears to be highly context dependent. While chronically elevated IL‐6 is commonly associated with systemic inflammation and disease‐related processes, transient increases in IL‐6 released from contracting skeletal muscle during exercise may exert anti‐inflammatory and metabolically beneficial effects through stimulation of anti‐inflammatory cytokines and suppression of pro‐inflammatory signalling pathways [S4]. Notably, SPARC, oncostatin M and decorin have received attention for their potential roles in cancer biology, including the regulation of tumour growth, extracellular matrix remodelling and anti‐tumour signalling [S5–S8]. These observations further support the concept that exercise‐induced skeletal muscle signalling may influence multiple physiological systems relevant to cancer outcomes.

**FIGURE 1:**
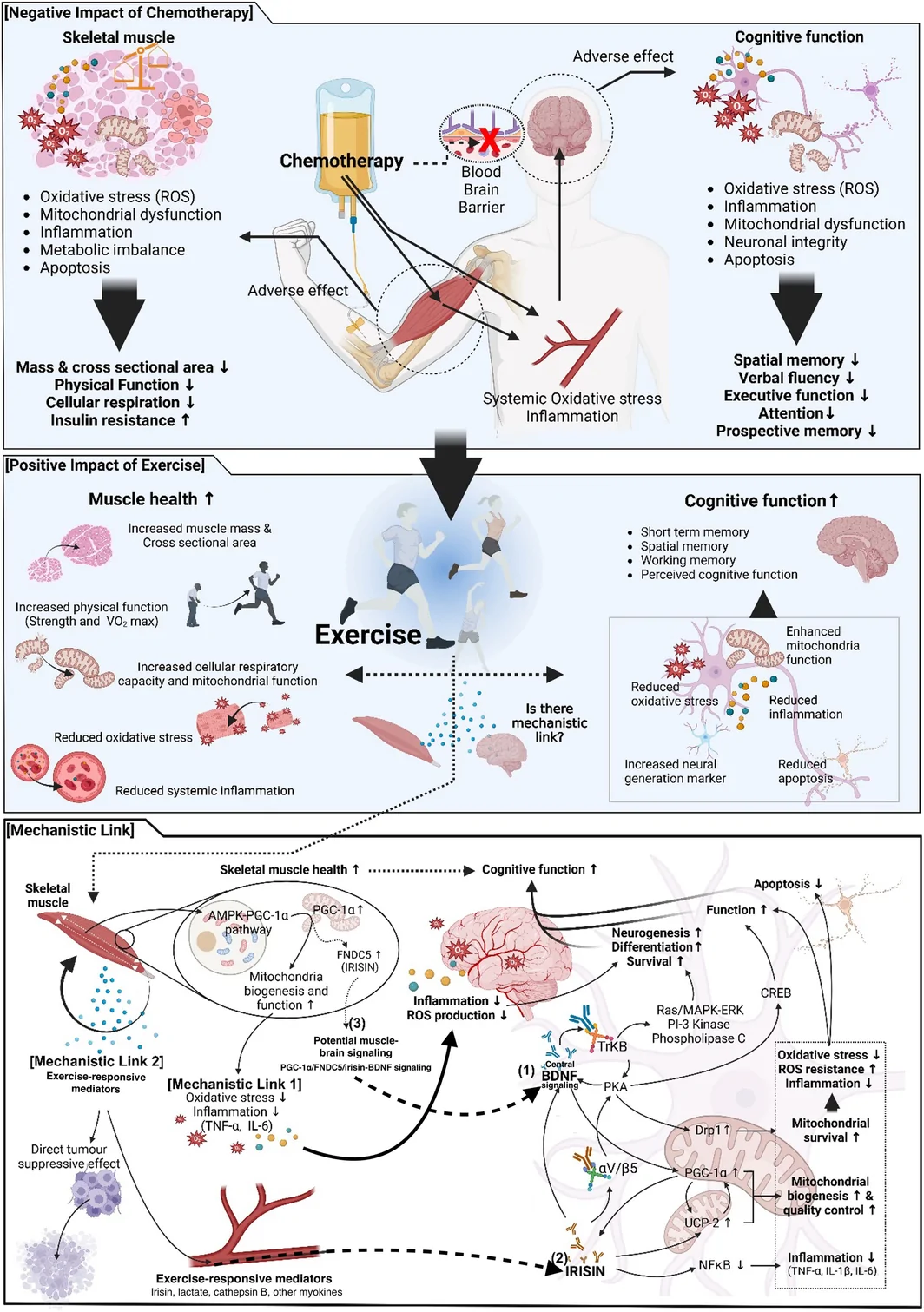
Overview of the relationship between chemotherapy‐induced skeletal muscle health reduction and cognitive function impairment, and potential mechanistic links between skeletal muscle health and cognitive function enhancement induced by exercise in patients undertaking chemotherapy. [Negative Impact of Chemotherapy] Chemotherapy reduces muscle mass and cross‐sectional area, decreases cellular respiration, increases insulin resistance and decreases physical function through mitochondrial dysfunction, metabolic imbalances, elevated oxidative stress, inflammation and apoptosis of muscle cells. The systemic oxidative stress and inflammatory cytokines generated by chemotherapy can induce severe oxidative stress and inflammation in neuronal cells. This leads to alterations in neuronal integrity and apoptosis, resulting in reduced spatial memory, verbal fluency, executive function, attention and prospective memory. [Positive Impact of Exercise] Exercise exerts its beneficial effects through multiple interconnected mechanisms, including improvements in mitochondrial function, reductions in oxidative stress and inflammation, metabolic adaptations and alterations in exercise‐responsive signalling mediators. Exercise enhances cellular respiratory capacity by improving mitochondrial function and lowering peripheral oxidative stress and inflammation, thereby promoting skeletal muscle health. Exercise has been shown to increase short‐term memory, spatial memory, working memory and perceived cognitive function by enhancing mitochondrial function in neurons and reducing oxidative stress and inflammation, which contribute to neuronal cell survival. [Mechanistic Link 1: Mitochondrial Function, Oxidative Stress and Inflammation] Exercise activates the AMPK‐PGC1α pathway in skeletal muscle and increases mitochondrial biogenesis and function, leading to reduced peripheral and systemic oxidative stress and inflammation. Lower levels of systemic oxidative stress and inflammation minimize the production of inflammatory markers and reactive oxygen species (ROS) in the brain. [Mechanistic Link 2: Exercise‐Responsive Mediators] Exercise induces a range of circulating and skeletal muscle‐derived signalling mediators, including irisin and other myokines, as well as non‐myokine mediators such as lactate and cathepsin B, which may contribute to muscle‐brain communication and brain‐related adaptations. These mediators are presented as components of a broader physiological network rather than as a single or exclusive pathway linking skeletal muscle and cognitive function. (1) Central BDNF signalling: BDNF binds to the TrkB receptor on neuronal cells, activating signalling pathways such as Ras/MAPK–ERK, PI3 Kinase and phospholipase C. This activation promotes neurogenesis, differentiation and the survival of neuronal cells. BDNF self‐regulates its signalling through the PKA pathway, enhancing the activity of Drp1 and PGC1α in neuronal cells and supporting mitochondrial survival, biogenesis and quality control. This reduces oxidative stress, ROS production and inflammation, enhancing neuronal function and reducing apoptosis. Central BDNF signalling is depicted separately from peripheral exercise‐responsive mediators, and the figure does not imply direct transport of circulating BDNF across the blood–brain barrier. (2) Irisin: Irisin binds to integrin αV/β5 on neuronal cells, increasing the expression of PKA and CREB. This promotes mitochondrial survival and function in neurons. Irisin mediates BDNF–TrkB signalling cascades (e.g., BDNF–TrkB‐MEK/ERK and BDNF‐PI3‐Akt). Additionally, irisin decreases ROS production and inflammation by increasing UCP‐2 expression in mitochondria and modulating NFκB signalling. Improved mitochondrial function and reduced inflammatory signalling reduce ROS production and inflammation, which contributes to enhanced neuronal function and reduced apoptosis. (3) Potential muscle‐brain signalling: Exercise‐induced activation of the AMPK‐PGC1α pathway increases the expression of PGC1α in skeletal muscle, which in turn increases FNDC5/irisin signalling. Peripheral exercise‐responsive signalling, including the PGC1α‐FNDC5/irisin pathway, has been proposed to influence BDNF expression and signalling within the brain. The PGC1α‐FNDC5/irisin‐BDNF signalling axis therefore represents one potential, rather than definitive, mechanism through which exercise may influence brain function.

Beyond myokines, other exercise‐responsive mediators have also been implicated in muscle–brain communication. For example, lactate has been shown to stimulate neurotrophic signalling pathways, including BDNF expression, and promote neuroplastic adaptations [S10], whereas cathepsin B released during exercise has been associated with enhanced hippocampal neurogenesis and memory function [S11]. These findings suggest that exercise‐induced brain adaptations are likely mediated through a complex network of interacting signalling molecules rather than a single pathway.

However, while these myokines and other exercise‐responsive mediators appear highly relevant to cancer biology and exercise adaptation, their direct relationships with cognitive function and muscle–brain communication remain incompletely characterized. In contrast, BDNF and irisin have received particular attention because of their established links to neurogenesis, neuronal survival, cognitive function and direct muscle–brain communication. Therefore, the following sections focus primarily on these two myokines as representative examples of exercise‐responsive mediators within the broader muscle–brain communication network [23, 24], [S3, S9].

BDNF is a key molecule involved in plastic changes in the brain, with its critical role in neuronal differentiation [S12], and direct crosstalk between the brain and skeletal muscle through BDNF is well described [119]. It is known that by binding with its key receptor, TrkB, BDNF can induce several cellular signalling cascades, such as Ras/MAPK–ERK, PI3‐K and PLC, critically impacting neuronal growth, maintenance, survival, as well as neuronal differentiation [S13]. Reduced levels of BDNF in the brain have been reported in other pathological conditions, such as Huntington's, Alzheimer's, Parkinson's disease and mild cognitive impairment [S12, S14], correlating to the severity of the disease [S12, S15]. Importantly, although the relationship between peripheral and central BDNF pools remains incompletely understood, circulating BDNF levels have been associated with cognitive function and synaptic health in both humans and animal models [S12, S16, S17]. Furthermore, exercise‐induced increases in peripheral BDNF may reflect broader neurobiological adaptations relevant to brain function, although the extent to which peripheral BDNF directly contributes to central BDNF signalling remains under investigation.

With its direct impact, BDNF positively modulates mitochondrial biogenesis and mitophagy [S18, S19]. In skeletal muscle, BDNF is produced and released by muscle contraction and elongation, and recent research demonstrated the role of BDNF in mitochondrial function and quality in a metabolically stressed environment [S18]. While hyperlipidaemia significantly increased inflammatory cytokines, including TNF‐α, IL‐6 and chemokine (C‐C motif) ligand‐2 and ‐5 in muscle‐specific bdnf knockout (MBKO) mice, BDNF mimetic (7,8‐dihydroxy flavone) increased mitochondrial content and quality in skeletal muscle through activation of the AMPK‐PGC‐1α signalling pathway, while also reducing inflammatory cytokine expression [S18]. This suggests BDNF is an essential myokine to maintain mitochondrial content and quality in skeletal muscle, which later can contribute to the improvement of systemic oxidative stress and inflammation [S3]. Indeed, metabolic stress induced by chemotherapy would likely be more profound than hyperlipidaemia. Nevertheless, the notion that BDNF could improve muscle mitochondria under metabolic stress provides positive insight into the function of exercise through its role in mitochondrial function and mitophagy. However, it should be emphasized that BDNF is unlikely to act independently of broader metabolic and inflammatory pathways. Rather, BDNF signalling appears to interact with mitochondrial quality control, oxidative stress regulation and inflammatory responses, which collectively influence skeletal muscle and brain health.

BDNF's positive role in mitochondria is not limited to skeletal muscle; BDNF is also a protective factor against ROS‐induced damage in neurons [S20, S21]. In preclinical studies, administration of BDNF alleviated the high ROS and H2O2‐induced cell death caused by 3‐nitropropionic acid in cortical cultures [S20, S21], improving neuronal survival [S3, S22]. In addition, BDNF can self‐regulate its signalling in neurons through TrkB‐dependent PKA activation [S23, S24]. PKA signalling is essential in maintaining mitochondrial homeostasis and inhibits the catalytic activity of Drp1 [S23, S24]. This can increase ROS resistance and improve mitochondrial stabilization and survival [S23, S24]. Moreover, BDNF stimulates mitochondrial biogenesis by enhancing PGC‐1α in neuronal mitochondria, which is critical for maintaining hippocampal dendritic spines and synapses [S25].

The cytokine irisin is another myokine receiving increased attention. Irisin is the cleaved product of transmembrane FNDC‐5, well‐known for providing favourable effects on metabolic diseases [S26]. Moreover, irisin can exert a beneficial effect on brain function by stimulating the cAMP/PKA/CREB pathway through its receptor integrin αV/β5 in neurons [S27]. With PKA, another signalling product, CREB is a cellular transcription factor that plays a crucial role in neuronal plasticity and long‐term memory formation [S13–S15]. Moreover, irisin mediates the irisin‐BDNF/TrKB‐MEK/ERK‐mTOR pathway and PI3/Akt/mTOR pathway, activating the STAT3 signalling pathway [S28, S29], which is confirmed to be correlated with stimulating hippocampal neurogenesis [S20, S30]. Importantly, a recent model presented by Pederson and colleagues, the Muscle–Brain Endocrine Loop, highlighted the direct role of irisin in cognitive function [S31]. It was postulated that the PGC1‐α/FNDC5(irisin)/BDNF pathway is tightly related in muscle–brain crosstalk [S31]. PGC‐1α expression is up‐regulated with FNDC5 gene expression in both neurons as well as skeletal muscle, and the increase of FNDC5 stimulated expression of BDNF in the brain [S32]. While irisin is a myokine that is a cleaved form of FNDC5, increased BDNF in the brain was shown by increased circulatory irisin and irisin knockout reduced BDNF expression in the brain [S33].

In addition to irisin's direct role in neurogenesis and maintenance, irisin also has anti‐oxidative effects. Reduced oxidative stress, increased muscular mitochondrial membrane potential and mitophagy were observed with irisin administration in an osteoarthritis animal model [S34]. In addition, in an ischemia/reperfusion liver animal model, irisin administration improved UCP‐2 expression, which is a key molecule for mitochondrial biogenesis, leading to reduced ROS production [S35, S36]. Collectively, these findings suggest that irisin may contribute to the regulation of oxidative stress, inflammation and mitochondrial homeostasis. Thus, its potential influence on cognitive outcomes is likely mediated through interactions with these broader physiological processes rather than through a direct isolated effect. A negative association between plasma irisin levels and brain infarct volume, neurological deficit and inflammation was also shown in an animal model, with reduced inflammation and oxidative stress through Akt and ERK1/2 pathway [S37]. Importantly, exogenous FNDC5 showed reduced ROS production, MDA level and NADPH oxidative activity in cardiac muscle in a doxorubicin‐treated animal model [S38]. Furthermore, the anti‐inflammatory role of irisin has been demonstrated in preclinical studies by showing the downregulation of the NFκB signalling pathway with reduced TNF‐α, IL‐1β and IL‐6, leading to reduced ROS production [S34, S39, S40]. Taken together, current evidence suggests that chemotherapy‐induced skeletal muscle dysfunction and cognitive impairment arise primarily from mitochondrial dysfunction, oxidative stress, inflammation and metabolic abnormalities [34, 35], [S41] Within this framework, alterations in myokine signalling may serve as one mechanism through which skeletal muscle communicates with the central nervous system. Therefore, BDNF and irisin should be viewed not as sole drivers of CICI, but as potential contributors within a multifactorial muscle–brain axis influenced by exercise and metabolic health.

## 6 Concluding Remarks—Clinical Implications and Future Perspectives

The growing recognition that skeletal muscle health influences systemic metabolism, inflammation and brain function has provided a new paradigm for targeted exercise medicine research and clinical practice [S42]. In line with this, we previously reviewed studies evaluating the tumour‐suppressive role of circulating myokines and suggested that more than 30 min of moderate‐ to high‐intensity aerobic exercise in addition to moderate‐ to high‐intensity resistance exercise is required to promote favourable physiological adaptations, including improvements in myokine signalling [S42]. However, further research considering specific conditions, such as cancer symptoms and treatment adverse effects, as well as biological mechanisms by which exercise influences cancer biology, is required for a more optimal exercise medicine prescription for patients with cancer [S42].

The blood–brain barrier prevents chemotherapeutic drugs from entering the brain [90], so CICI as a direct effect is not logical and has been described as a ‘bizarre pattern’ [S42]. Moreover, patients receiving chemotherapy are likely to have undertaken or are undertaking different types of cancer treatments; thus, there are challenges in implementing treatment strategies to alleviate and prevent CICI. Recently, a feedback loop of chemotherapy‐induced peripheral oxidative stress and inflammation creating severe endogenous inflammation and oxidative stress in the hippocampal area profoundly impacting cognitive function has been revealed and provides a pathway forward [19, 20, 21]. Furthermore, it has also been proposed that the non‐specific action of chemotherapy impairing muscle mitochondrial function causes peripheral oxidative stress and inflammation [47, 48], highlighting skeletal muscle as a potential therapeutic target for patients experiencing or at risk of developing CICI.

Indeed, exercise is currently the most consistently effective intervention for improving skeletal muscle health without adverse effects in patients with cancer [33], [S43‐S45], and exercise‐induced improvements in skeletal muscle health are likely mediated through multiple adaptations, including enhanced mitochondrial function, reduced oxidative stress and inflammation, improved metabolic regulation and alterations in endocrine signalling [22, 23]. Among the various adaptations induced by exercise, improvements in mitochondrial function, reductions in oxidative stress and inflammation and alterations in myokine signalling (including BDNF and irisin) may collectively contribute to improved cognitive outcomes [22, 23, 24], [S3, S26, S31]. However, as routine myokine and mitochondrial function evaluation are nearly impossible to implement in exercise medicine, careful attention should be given to VO_2_ max. As there is a linear relationship between VO_2_ max and muscle mitochondrial content [116], enhanced VO_2_ max may represent improved muscle anti‐oxidative capacity resulting from enhanced muscle mitochondrial content [S46]. Moreover, exercise has been shown to improve VO_2_ max and cognitive function in patients undertaking chemotherapy (Table 2) and persons without cancer (Table 3), suggesting a link between exercise‐induced adaptations, specifically on mitochondria and cognitive function. Importantly, different exercise modalities may contribute through distinct physiological mechanisms. Aerobic exercise primarily improves cardiorespiratory fitness, mitochondrial function, insulin sensitivity and oxidative stress regulation, whereas resistance exercise plays a critical role in preserving skeletal muscle mass and strength by stimulating anabolic pathways and attenuating muscle protein breakdown. Therefore, both exercise modalities may provide complementary benefits for patients undergoing chemotherapy.

**TABLE 3:** Effect of exercise on muscle and cognitive function in older patients: Clinical evidence.

Author	Population	Exercise	Muscular improvements	Cognitive improvements	Additional
Baker et al., 2010 [S47]	Older adults with mild cognitive impairment (*n* = 33) Control (*n* = 10); exercise (*n* = 23)	24‐week high‐intensity aerobic exercise (45–60 min/day for 4 days/week; high intensity 75%–85% HRR)	Body fat ↓; total cholesterol level ↓; VO_2_peak ↑; treadmill grade ↑; treadmill time to exhaustion ↑; Women: total fat ↓	Symbol digit modalities ↑; verbal frequency ↑; trail B time ↓; task switching ↔; women: stroop test ↓	Total cholesterol level ↓; LDL ↓; HDL ↓; TG ↔; women: glucose regulation ↑, insulin sensitivity ↑, VO_2_ peak positively associated with executive function; men: plasma BDNF less decrease
Yoon et al., 2017 [S48]	Older adults with mild cognitive impairment (*n* = 58) Control (*n* = 19); low speed strength training (*n* = 19); high speed power training (*n* = 20)	12‐week exercise with elastic bands (60 min/day for 2 days/week; high speed: low‐intensity band, low speed: high‐intensity band, RPE 15–16)	Low speed: body weight ↑, SPPB ↑; High speed: body weight ↓, BMI ↓, skeletal muscle mass ↑, percent body fat ↓, waist‐hip ratio ↓, SPPB ↑, grip strength ↑	Low speed: MMSE ↑; high speed: MMSE ↑, MoCA ↑; control MMSE ↓	High speed: SBP ↓
Yoon et al., 2018 [S49]	Frail older adult with dementia (*n* = 66) Control (*n* = 33); exercise (*n* = 33)	16‐Week high‐speed resistance training with elastic bands (60 min/day for 3 days/week; moderate intensity 12–13 RPE)	SPPB ↑; time up and go ↓; gait speed ↓; grip strength ↑; isokinetic knee strength and endurance ↑	Processing speed: trail making test part A ↓, frontal assessment battery ↑	—
Langoni et al., 2019 [S50]	Sedentary older adults with mild cognitive impairment (*n* = 52) control (*n* = 26); intervention (*n* = 26)	24‐week aerobic + resistance exercise (60 min/day for 2 days/week)	Stationary walk test ↑; sit and stand test ↑; functional reach test ↑	MMSE ↑	—
Thapa et al., 2022 [S51]	Middle‐aged women (*n* = 48) Electrical muscle stimulation with resistance exercise (ERT, *n* = 16); resistance exercise (RT, *n* = 16); control (CON, *n* = 16)	4‐week leg resistance exercise with and without EMS (50 min/day for 3 days/week)	ERT: skeletal muscle mass index ↑, phase angle ↑, gait speed ↑, handgrip strength ↑, 5 min sit‐to‐stand test ↓, time up and go ↓; RT: time up and go ↓	ERT: resting beta 1 band power ↑, resting theta 1 band power↓, alpha 1 band connectivity ↑	—
Tan et al., 2023 [S52]	Pre‐frail older adults (*n* = 140) Control (*n* = 95); exercise (EX, *n* = 26); exercise + psychology program (Ex+CST, *n* = 19)	24‐week aerobic + resistance + balance + dual task program (60 min/day for 2 days/week)	3‐month: gait speed ↑, SPPB ↑, 5x‐STS ↓; 6‐month: gait speed ↑, FRIAL total ↓, appendicular skeletal muscle mass index ↑; body cell mass ↑, phase angle ↑; 12‐month: gait speed ↑, less decline of appendicular skeletal muscle mass index	3‐month: MoCA ↑; 6‐month: MoCA ↑	3‐month: TNF‐α ↓, geriatric depression symptom ↓; 6‐month: geriatric depression symptom ↓; 12‐month: perceived health ↑
Kim et al., 2015 [S53]	19 months old male C57BL/6 mice (*n* = 13) Control (*n* = 6); exercise (*n* = 7)	12‐week ladder climbing (1 m ladder for 3 days/week)	Grip strength ↑; muscle quality ↑; mobility ↑	—	Serum irisin ↑; soleus muscle irisin ↑
	Women aged over 65 years (*n* = 40) control (*n* = 14); exercise (*n* = 26)	12‐week elastic band‐based resistance exercise (60 min/week for 2 days/week)	Isokinetic: knee extension strength ↑, keen flexion strength ↑, knee extension endurance ↑, knee flexion endurance ↑; grip strength ↑	—	Irisin ↑ (positive correlation with grip strength and leg strength)
Miyamoto‐Mikami et al., 2015 [S54]	Young (*n* = 25) and older adults (*n* = 28) young control (*n* = 11); young exercise (=14); older control (*n* = 14); older exercise (*n* = 14)	8‐Week cycling (55 min/day for 3 days/week; 60%–70% VO_2_ peak)	Young: BMI ↓, body weight ↓, fat percentage ↓, whole body fat mass ↓, abdominal subcutaneous adipose tissue ↓, VO_2_ peak ↑; old: fat percentage ↓, whole body fat mass ↓, arm fat mass ↓, abdominal visceral adipose tissue ↓, VO_2_ peak ↑	—	Serum irisin ↑; young ↑ (vs old), old exercise ↑ (vs old control)
Jürimäe et al., 2023 [S55]	Older men (*n* = 11)	3‐Week sprint interval training (6 reps of 30‐s all‐out cycling +4 min rest for 3 days/week)	VO_2_ peak ↑	—	Irisin ↑, IL‐6 ↓, TNF‐α ↓
Song et al., 2019 [S56]	Older adults with mild cognitive impairment (*n* = 120) Exercise (*n* = 60); control (*n* = 60)	16‐Week aerobic stepping exercise (60 min/day for 3 days/week; moderate intensity RPE 12–14)	—	MoCA ↑; quality of life for Alzheimer's disease ↑	Geriatric depression symptom ↓; Pittsburgh Sleep Quality Index ↓
Tsai et al., 2019 [S57]	Older adults with amnestic mild cognitive impairment (*n* = 55) control (*n* = 18); aerobic (*n* = 19); resistance (*n* = 18)	16‐Week supervised aerobic (40 min/day for 3 days/week, 50%–60% to 70%–75% HRR) 16‐Week supervised resistance (40 min/day for 3 days/week, 60%–70% of 1RM to 75% of 1RM, 3 sets of 10 reps)	Cardiorespiratory fitness ↑ (aerobic group)	Aerobic: accuracy rates ↑, reaction times ↓, event‐related potential P3 amplitude ↑; resistance: accuracy rates ↑, reaction times ↓, event‐related potential P3 amplitude ↑	Aerobic: Peripheral BDNF ↑, insulin ↓, TNF‐a ↓, IL‐15 ↓; resistance: insulin ↓, IGF‐1 ↑, IL‐15 ↓; control: TNF‐α ↑
Asteasu et al., 2021 [S58]	Hospitalized elderly (*n* = 370) exercise (*n* = 185); control (*n* = 185)	5–7 days aerobic + resistance exercise (20 min for 2 session/day: supervised in morning + home‐based in evening)	SPPB ↑	MMSE ↑; verbal fluency ability score (regression) ↑	—

*Note:* The presented results are all significant (*p* < 0.05), and the increases and reductions are presented with arrows (↑ and ↓).

Abbreviations: 5x‐STS, 5 times sit to stand; BDNF, Brain‐derived neurotrophic factor; BMI, body mass index; HDL, high‐density lipoprotein; HRR, heart rate reserve; IGF‐1, insulin‐like growth factor‐1; IL‐6, interleukin‐ 6; IL‐15, interleukin‐15; LDL, low‐density lipoprotein; MMSE, Mini‐Mental State Examination; MoCA, Montreal Cognitive Assessment; RM, repetition maximum; RPE, rate of perceived exertion; SBP, systolic blood pressure; SPPB, Short Physical Performance Battery; TNF‐α, tumour necrosis factor‐alpha; VO_2_peak, peak oxygen consumption.

However, most clinical exercise trials in patients with chemotherapy implemented mixed‐mode (resistance and aerobic) exercise, making it challenging to differentiate exercise‐type effects. The exercise trials summarized in Table 3 have demonstrated substantial improvements in aerobic fitness and cognitive function, with some studies also reporting favourable changes in circulating myokines and mitochondrial‐related adaptations [S47‐S58]. Moreover, animal studies with various chemotherapy models also showed positive effects of aerobic exercise (e.g., treadmill exercise) on cognitive function while improving muscle mitochondrial function—anti‐oxidative capacity and systemic inflammation [72, 107, 108]. Importantly, from our previous research, we have reported improved circulating myokine levels in patients with advanced prostate cancer after long‐term exercise despite the removal of essential signalling for muscle hypertrophy (androgen deprivation therapy) [S59‐S61], suggesting that myokine synthesis and secretion may not be entirely dependent on skeletal muscle hypertrophy signalling.

As such, when prescribing exercise medicine for patients undergoing chemotherapy who are experiencing or at risk of CICI, improving cardiorespiratory fitness (e.g., VO_2_max) should be considered an important therapeutic target, while preservation of skeletal muscle mass and function should also remain a key clinical objective. Based on findings in older adults (Table 3), potentially beneficial aerobic exercise may consist of ~60 min of continuous moderate‐intensity exercise (60%–70% of VO_2_max) or 30–40 min of high‐intensity interval exercise, with peak‐intensity bouts at 70%–80% of VO_2_max.

Further, resistance exercise is also known to have a positive impact on cancer outcomes (e.g., progression, disease‐free survival and overall survival), as well as treatment‐related adverse effects (e.g., fatigue, reduced skeletal muscle mass, increased fat mass, reduced quality of life) [33], [S43, S44]. While aerobic exercise may be particularly important for improving mitochondrial health and reducing systemic oxidative stress and inflammation, resistance exercise remains essential for counteracting treatment‐related muscle loss and functional decline. Thus, it has been advised that resistance exercise should also be prescribed, preferably every second day, accompanying aerobic exercise to provide collective benefits in this patient group. Although evidence on combined exercise modalities in patients with cancer remains limited, integrating resistance exercise with aerobic components may be a promising approach for individuals experiencing or at risk of CICI. Indirect support comes from studies in older adults with cognitive impairment, where modified resistance modalities such as high‐speed power training (low load, high‐repetition exercise emphasizing contraction speed) have been associated with improvements in both cognitive function and skeletal muscle mass [S48–50, S58]. These findings suggest that exploring modified resistance exercise strategies in cancer populations may be warranted, particularly for patients at risk of cognitive decline.

Moreover, future translational research should investigate the relative contributions of mitochondrial adaptations, oxidative stress reduction, inflammatory regulation and myokine signalling to exercise‐induced improvements in cognitive function during chemotherapy. Such research would provide further information about effective and efficient exercise medicine that could provide collective benefits through improvements in skeletal muscle health, metabolic function and brain health—enhanced survival (skeletal muscle health improvement) and quality of life (cognitive function improvement)—in this patient group.

In summary, current evidence suggests that CICI is unlikely to result from a single biological pathway. Rather, mitochondrial dysfunction, oxidative stress, systemic inflammation and metabolic abnormalities appear to represent central mechanisms affecting both skeletal muscle and brain health [38, 39]. Within this broader framework, exercise‐induced myokines such as BDNF and irisin may contribute to muscle–brain communication and represent one potential mechanism through which exercise exerts neuroprotective effects. Future studies should clarify the relative contribution of these interconnected pathways to optimize exercise medicine strategies for patients undergoing chemotherapy.

## Nomenclature

AMPK: AMP‐activated protein kinase
BDNF: brain‐derived neurotrophic factor
CREB: cAMP response element‐binding protein
DNA: deoxyribonucleic acid
Drp1: dynamin‐related protein 1
DTI: diffusion tensor imaging
ERK2: extracellular signal‐regulated kinase 2
FACT‐Cog: functional assessment of cancer treatment—cognition
FADH_2_
: flavin adenine dinucleotide
Fbxo21: F‐box protein 21
Fbxo30: F‐box protein 30
Fbxo31: F‐box protein 31
FGF‐21: fibroblast growth factor 21
FNDC‐5: fibronectin type III domain‐containing protein‐5
GPX1: glutathione peroxidase 1
GSH: glutathione
H^1^‐MRS: proton magnetic resonance spectroscopy
IFN‐γ: interferon‐γ
IL‐15: interleukin‐15
IL‐1ra: interleukin‐1 receptor antagonist
IL‐1β: interleukin‐1 beta
IL‐6: interleukin‐6
MDA: malondialdehyde
Mfn1: mitofusin 1
Mfn2: mitofusin 2
MIP‐1α: macrophage inflammatory protein‐1 alpha
Mn‐SOD: manganese‐superoxide dismutase
MRS: multivoxel magnetic resonance spectroscopy
mtDNA: mitochondrial DNA
MuRF‐1: muscle ring‐finger protein‐1
NAA: N‐acetylaspartate
NADH: nicotinamide adenine dinucleotide
NADPH: nicotinamide adenine dinucleotide phosphate
NFκB: nuclear factor kappa B
PGC‐1α: peroxisome proliferator‐activated receptor gamma coactivator 1‐alpha
PGC‐1β: peroxisome proliferator‐activated receptor gamma coactivator 1‐beta
PI3‐K: phosphoinositide 3‐kinase
PINK1: PTEN‐induced putative kinase 1
PKA: protein kinase A
PLC: phospholipase C
PRDX1: peroxiredoxin 1
protein‐bound HNE: protein‐bound 4‐hydroxynonenal
PTP: permeability transition pore
ROS: reactive oxygen species
SIRT: sirtuin
SOD: superoxide dismutase
SPARC: secreted protein acidic and rich in cysteine
TFAM: mitochondrial transcription factor A
TNF‐α: tumour necrosis factor‐alpha
TrkB: tropomyosin receptor kinase B
TUNEL: terminal deoxynucleotidyl transferase dUTP nick‐end labelling
VDAC: voltage‐dependent anion channel
VO_2_ max: maximal oxygen consumption

## Author Contributions

Conceptualization, S.Y.A., J.‐S.K., R.U.N, and W.S.; investigation, S.Y.A. and J.‐S.K.; writing – original draft, S.Y.A. and J.‐S.K.; writing – review and editing, S.Y.A., J.‐S.K., R.U.N., D.A.G., D.R.T., W.S., D.H.Y., C.M., C.S., Y.J.C., and J.M.; Supervision, R.U.N., D.A.G., D.R.T.

## Funding

The authors have nothing to report.

## Conflicts of Interest

The authors declare no conflicts of interest.

## Supporting information


**Data S1:** Chemotherapy induced skeletal muscle dysfunction and cognitive impairment.

## Data Availability

Data sharing not applicable to this article as no datasets were generated or analysed during the current study.
