# Supplementary material for: Chemotherapy‐Induced Skeletal Muscle Dysfunction and Cognitive Impairment: A Muscle‐Brain Mechanistic Framework With Implications for Exercise Oncology
Source: J Cachexia Sarcopenia Muscle. 2026 Oct 7;17(5):e70394. doi: 10.1002/jcsm.70394 (PMC13643939; doi:10.1002/jcsm.70394)
Supplement: Supplementary file 1 [file JCSM-17-e70394-s001.docx]

**Supporting Information**

**Additional References**

S1. Leek BT, Mudaliar SR, Henry R, Mathieu-Costello O, Richardson RS. Effect of acute exercise on citrate synthase activity in untrained and trained human skeletal muscle. American Journal of Physiology-Regulatory, Integrative and Comparative Physiology. 2001;280(2):R441-7.
S2. Menshikova EV, Ritov VB, Fairfull L, Ferrell RE, Kelley DE, Goodpaster BH. Effects of exercise on mitochondrial content and function in aging human skeletal muscle. The Journals of Gerontology, Series A: Biological Sciences and Medical Sciences. 2006;61(6):534-40.
S3. Pang BPS, Chan WS, Chan CB. Mitochondria homeostasis and oxidant/antioxidant balance in skeletal muscle-do myokines play a role? Antioxidants (Basel). 2021;10(2).
S4. Pedersen BK, Febbraio MA. Muscle as an endocrine organ: focus on muscle-derived interleukin-6. Physiological Reviews. 2008;88(4):1379-406.
S5. Aoi W, Naito Y, Takagi T, Tanimura Y, Takanami Y, Kawai Y, et al. A novel myokine, secreted protein acidic and rich in cysteine (SPARC), suppresses colon tumorigenesis via regular exercise. Gut. 2013;62(6):882-9.
S6. Liu YP, Hsiao M. Exercise-induced SPARC prevents tumorigenesis of colon cancer. Gut. 2013;62(6):810-1.
S7. Hojman P, Dethlefsen C, Brandt C, Hansen J, Pedersen L, Pedersen BK. Exercise-induced muscle-derived cytokines inhibit mammary cancer cell growth. American Journal of Physiology-Endocrinology and Metabolism. 2011;301(3):E504-10.
S8. Kanzleiter T, Rath M, Görgens SW, Jensen J, Tangen DS, Kolnes AJ, et al. The myokine decorin is regulated by contraction and involved in muscle hypertrophy. Biochemical and Biophysical Research Communications. 2014;450(2):1089-94.
S9. Schnyder S, Handschin C. Skeletal muscle as an endocrine organ: PGC-1α, myokines and exercise. Bone. 2015;80:115-25.
S10. El Hayek L, Khalifeh M, Zibara V, Abi Assaad R, Emmanuel N, Karnib N, et al. Lactate mediates the effects of exercise on learning and memory through SIRT1-dependent activation of hippocampal brain-derived neurotrophic factor (BDNF). Journal of Neuroscience. 2019;39(13):2369-82.
S11. Moon HY, Becke A, Berron D, Becker B, Sah N, Benoni G, et al. Running-induced systemic cathepsin B secretion is associated with memory function. Cell Metabolism. 2016;24(2):332-40.
S12. Miranda M, Morici JF, Zanoni MB, Bekinschtein P. Brain-derived neurotrophic factor: a key molecule for memory in the healthy and the pathological brain. Frontiers in Cellular Neuroscience. 2019;13:363.
S13. Reichardt LF. Neurotrophin-regulated signalling pathways. Philosophical Transactions of the Royal Society B: Biological Sciences. 2006;361(1473):1545-64.
S14. Shimada H, Makizako H, Doi T, Yoshida D, Tsutsumimoto K, Anan Y, et al. A large, cross-sectional observational study of serum BDNF, cognitive function, and mild cognitive impairment in the elderly. Frontiers in Aging Neuroscience. 2014;6:69.
S15. Peng S, Wuu J, Mufson EJ, Fahnestock M. Precursor form of brain-derived neurotrophic factor and mature brain-derived neurotrophic factor are decreased in the pre-clinical stages of Alzheimer's disease. Journal of Neurochemistry. 2005;93(6):1412-21.
S16. Intlekofer KA, Cotman CW. Exercise counteracts declining hippocampal function in aging and Alzheimer's disease. Neurobiology of Disease. 2013;57:47-55.
S17. Morse JK, Wiegand SJ, Anderson K, You Y, Cai N, Carnahan J, et al. Brain-derived neurotrophic factor (BDNF) prevents the degeneration of medial septal cholinergic neurons following fimbria transection. Journal of Neuroscience. 1993;13(10):4146-56.
S18. Ahuja P, Ng CF, Pang BPS, Chan WS, Tse MCL, Bi X, et al. Muscle-generated BDNF (brain derived neurotrophic factor) maintains mitochondrial quality control in female mice. Autophagy. 2022;18(6):1367-84.
S19. S KS, Swain M, Dagda RK. BDNF-TrkB signaling in mitochondria: implications for neurodegenerative diseases. Molecular Neurobiology. 2024.
S20. Molinari C, Morsanuto V, Ruga S, Notte F, Farghali M, Galla R, et al. The role of bdnf on aging-modulation markers. Brain Sciences. 2020;10(5).
S21. Wu CL, Chen SD, Yin JH, Hwang CS, Yang DI. Nuclear factor-kappaB-dependent sestrin2 induction mediates the antioxidant effects of BDNF against mitochondrial inhibition in rat cortical neurons. Molecular Neurobiology. 2016;53(6):4126-42.
S22. Petzold A, Psotta L, Brigadski T, Endres T, Lessmann V. Chronic BDNF deficiency leads to an age-dependent impairment in spatial learning. Neurobiology of Learning and Memory. 2015;120:52-60.
S23. Merrill RA, Strack S. Mitochondria: a kinase anchoring protein 1, a signaling platform for mitochondrial form and function. The International Journal of Biochemistry & Cell Biology. 2014;48:92-6.
S24. Swain M, S KS, Tapia K, Dagda RY, Dagda RK. Brain-derived neurotrophic factor protects neurons by stimulating mitochondrial function through protein kinase A. Journal of Neurochemistry. 2023;167(1):104-25.
S25. Cheng A, Wan R, Yang JL, Kamimura N, Son TG, Ouyang X, et al. Involvement of PGC-1α in the formation and maintenance of neuronal dendritic spines. Nature Communication. 2012;3:1250.
S26. Novelle MG, Contreras C, Romero-Pico A, Lopez M, Dieguez C. Irisin, two years later. International Journal of Endocrinology. 2013;2013:746281.
S27. Jackson TC, Gorse K, Herrmann JR, Kochanek PM. Hippocampal and prefrontal cortical brain tissue levels of irisin and gdf15 receptor subunits in children. Molecular Neurobiology. 2021;58(5):2145-57.
S28. Collo G, Cavalleri L, Spano P. Structural plasticity in mesencephalic dopaminergic neurons produced by drugs of abuse: critical role of BDNF and dopamine. Frontiers in Pharmacology. 2014;5:259.
S29. Moon HS, Dincer F, Mantzoros CS. Pharmacological concentrations of irisin increase cell proliferation without influencing markers of neurite outgrowth and synaptogenesis in mouse H19-7 hippocampal cell lines. Metabolism. 2013;62(8):1131-6.
S30. Jung KH, Chu K, Lee ST, Kim SJ, Sinn DI, Kim SU, et al. Granulocyte colony-stimulating factor stimulates neurogenesis via vascular endothelial growth factor with STAT activation. Brain Research. 2006;1073-1074:190-201.
S31. Pedersen BK. Physical activity and muscle-brain crosstalk. Nature Reviews Endocrinology. 2019;15(7):383-92.
S32. Boström P, Wu J, Jedrychowski MP, Korde A, Ye L, Lo JC, et al. A PGC1-α-dependent myokine that drives brown-fat-like development of white fat and thermogenesis. Nature. 2012;481(7382):463-8.
S33. Severinsen MCK, Pedersen BK. Muscle-organ crosstalk: the emerging roles of myokines. Endocrine Review. 2020;41(4):594-609.
S34. Wang FS, Kuo CW, Ko JY, Chen YS, Wang SY, Ke HJ, et al. Irisin mitigates oxidative stress, chondrocyte dysfunction and osteoarthritis development through regulating mitochondrial integrity and autophagy. Antioxidants (Basel). 2020;9(9).
S35. Bi J, Yang L, Wang T, Zhang J, Li T, Ren Y, et al. Irisin improves autophagy of aged hepatocytes via increasing telomerase activity in liver injury. Oxidative Medicine and Cellular Longevity. 2020;2020:6946037.
S36. Bi J, Zhang J, Ren Y, Du Z, Li Q, Wang Y, et al. Irisin alleviates liver ischemia-reperfusion injury by inhibiting excessive mitochondrial fission, promoting mitochondrial biogenesis and decreasing oxidative stress. Redox Biology. 2019;20:296-306.
S37. Li DJ, Li YH, Yuan HB, Qu LF, Wang P. The novel exercise-induced hormone irisin protects against neuronal injury via activation of the Akt and ERK1/2 signaling pathways and contributes to the neuroprotection of physical exercise in cerebral ischemia. Metabolism. 2017;68:31-42.
S38. Zhang X, Hu C, Kong CY, Song P, Wu HM, Xu SC, et al. FNDC5 alleviates oxidative stress and cardiomyocyte apoptosis in doxorubicin-induced cardiotoxicity via activating AKT. Cell Death & Differentiation. 2020;27(2):540-55.
S39. Mazur-Bialy AI, Bilski J, Pochec E, Brzozowski T. New insight into the direct anti-inflammatory activity of a myokine irisin against proinflammatory activation of adipocytes. Implication for exercise in obesity. Journal of Physiology and Pharmacology. 2017;68(2):243-51.
S40. Peng J, Deng X, Huang W, Yu JH, Wang JX, Wang JP, et al. Irisin protects against neuronal injury induced by oxygen-glucose deprivation in part depends on the inhibition of ROS-NLRP3 inflammatory signaling pathway. Molecular Immunology. 2017;91:185-94.
S41. Ahles TA, Root JC. Cognitive effects of cancer and cancer treatments. Annual Review of Clinical Psychology. 2018;14:425-51.
S42. Kim J-S, Galvão DA, Newton RU, Gray E, Taaffe DR. Exercise-induced myokines and their effect on prostate cancer. Nature Reviews Urology. 2021;18(9):519-42.
S43. Campbell KL, Winters-Stone KM, Wiskemann J, May AM, Schwartz AL, Courneya KS, et al. Exercise guidelines for cancer survivors: consensus statement from international multidisciplinary roundtable. Medicine & Science in Sports & Exercise. 2019;51(11):2375-90.
S44. Hayes SC, Newton RU, Spence RR, Galvão DA. The exercise and sports science australia position statement: exercise medicine in cancer management. Journal of Science and Medicine in Sport. 2019; 22(11), 1175-99.
S45. Rock CL, Thomson C, Gansler T, Gapstur SM, McCullough ML, Patel AV, et al. American Cancer Society guideline for diet and physical activity for cancer prevention. CA: A Cancer Journal for Clinicians. 2020;70(4):245-71.
S46. Lundby C, Montero D, Joyner M. Biology of VO(2) max: looking under the physiology lamp. Acta Physiologica (Oxford, England). 2017;220(2):218-28.
S47. Baker LD, Frank LL, Foster-Schubert K, Green PS, Wilkinson CW, McTiernan A, et al. Effects of aerobic exercise on mild cognitive impairment: a controlled trial. Archives of Neurology. 2010;67(1):71-9.
S48. Yoon DH, Kang D, Kim HJ, Kim JS, Song HS, Song W. Effect of elastic band-based high-speed power training on cognitive function, physical performance and muscle strength in older women with mild cognitive impairment. Geriatrics & Gerontology International. 2017;17(5):765-72.
S49. Yoon DH, Lee JY, Song W. Effects of resistance exercise training on cognitive function and physical performance in cognitive frailty: a randomized controlled trial. The Journal of Nutrition, Health and Aging. 2018;22(8):944-51.
S50. Langoni CDS, Resende TL, Barcellos AB, Cecchele B, Knob MS, Silva TDN, et al. Effect of exercise on cognition, conditioning, muscle endurance, and balance in older adults with mild cognitive impairment: a randomized controlled trial. Journal of Geriatric Physical Therapy. 2019;42(2):E15-e22.
S51. Thapa N, Yang JG, Bae S, Kim GM, Park HJ, Park H. Effect of electrical muscle stimulation and resistance exercise intervention on physical and brain function in middle-aged and older women. International Journal of Environmental Research and Public Health. 2022;20(1).
S52. Tan LF, Chan YH, Seetharaman S, Denishkrshna A, Au L, Kwek SC, et al. Impact of exercise and cognitive stimulation therapy on physical function, cognition and muscle mass in pre-frail older adults in the primary care setting: a cluster randomized controlled trial. The Journal of nutrition, health and aging. 2023;27(6):438-47.
S53. Kim HJ, So B, Choi M, Kang D, Song W. Resistance exercise training increases the expression of irisin concomitant with improvement of muscle function in aging mice and humans. Experimental Gerontology. 2015;70:11-7.
S54. Miyamoto-Mikami E, Sato K, Kurihara T, Hasegawa N, Fujie S, Fujita S, et al. Endurance training-induced increase in circulating irisin levels is associated with reduction of abdominal visceral fat in middle-aged and older adults. PLoS One. 2015;10(3):e0120354.
S55. Jürimäe J, Purge P, Remmel L, Ereline J, Kums T, Kamandulis S, et al. Changes in irisin, inflammatory cytokines and aerobic capacity in response to three weeks of supervised sprint interval training in older men. The Journal of Sports Medicine and Physical Fitness. 2023;63(1):162-9.
S56. Song D, Yu DSF. Effects of a moderate-intensity aerobic exercise programme on the cognitive function and quality of life of community-dwelling elderly people with mild cognitive impairment: A randomised controlled trial. International Journal of Nursing Studies. 2019;93:97-105.
S57. Tsai CL, Pai MC, Ukropec J, Ukropcová B. Distinctive effects of aerobic and resistance exercise modes on neurocognitive and biochemical changes in individuals with mild cognitive impairment. Current Alzheimer Research. 2019;16(4):316-32.
S58. Sáez de Asteasu ML, Martínez-Velilla N, Zambom-Ferraresi F, Ramírez-Vélez R, García-Hermoso A, Izquierdo M. Cognitive function improvements mediate exercise intervention effects on physical performance in acutely hospitalized older adults. Journal of the American Medical Directors Association. 2021;22(4):787-91.
S59. Kim J-S, Taaffe DR, Galvão DA, Clay TD, Redfern AD, Hart NH, et al. Acute effect of high-intensity interval aerobic exercise on serum myokine levels and resulting tumour-suppressive effect in trained patients with advanced prostate cancer. Prostate Cancer and Prostatic Diseases. 2022.
S60. Kim, J. S., Taaffe, D. R., Galvão, D. A., Saad, F., & Newton, R. U. (2023). Exercise Mediates Myokine Release and Tumor Suppression in Prostate Cancer Independent of Androgen Signaling. Exercise and sport sciences reviews, *51*(4), 161-168. https://doi.org/10.1249/JES.0000000000000323
S61. Kim JS, Taaffe DR, Galvão DA, Hart NH, Gray E, Ryan CJ, et al. Exercise in advanced prostate cancer elevates myokine levels and suppresses in-vitro cell growth. Prostate Cancer and Prostatic Diseases. 2022:1-7.
